# Microorganisms oxidize glucose through distinct pathways in permeable and cohesive sediments

**DOI:** 10.1093/ismejo/wrae001

**Published:** 2024-01-30

**Authors:** Tess F Hutchinson, Adam J Kessler, Wei Wen Wong, Puspitaningsih Hall, Pok Man Leung, Thanavit Jirapanjawat, Chris Greening, Ronnie N Glud, Perran L M Cook

**Affiliations:** Water Studies, School of Chemistry, Monash University, Clayton, VIC 3800, Australia; School of Earth, Atmosphere & Environment, Monash University, Clayton, VIC 3800, Australia; Water Studies, School of Chemistry, Monash University, Clayton, VIC 3800, Australia; Water Studies, School of Chemistry, Monash University, Clayton, VIC 3800, Australia; Department of Microbiology, Biomedicine Discovery Institute, Monash University, Clayton, VIC 3800, Australia; Department of Microbiology, Biomedicine Discovery Institute, Monash University, Clayton, VIC 3800, Australia; Department of Microbiology, Biomedicine Discovery Institute, Monash University, Clayton, VIC 3800, Australia; University of Southern Denmark, HADAL, Nordcee and DIAS, Odense M 5230, Denmark; Tokyo University of Marine Science and Technology, 4-5-7 Konan, Minato-ku, Tokyo 108-8477, Japan; Water Studies, School of Chemistry, Monash University, Clayton, VIC 3800, Australia

**Keywords:** fermentation, respiration, organic carbon, permeable sediments

## Abstract

In marine sediments, microbial degradation of organic matter under anoxic conditions is generally thought to proceed through fermentation to volatile fatty acids, which are then oxidized to CO_2_ coupled to the reduction of terminal electron acceptors (e.g. nitrate, iron, manganese, and sulfate). It has been suggested that, in environments with a highly variable oxygen regime, fermentation mediated by facultative anaerobic bacteria (uncoupled to external terminal electron acceptors) becomes the dominant process. Here, we present the first direct evidence for this fermentation using a novel differentially labeled glucose isotopologue assay that distinguishes between CO_2_ produced from respiration and fermentation. Using this approach, we measured the relative contribution of respiration and fermentation of glucose in a range of permeable (sandy) and cohesive (muddy) sediments, as well as four bacterial isolates. Under anoxia, microbial communities adapted to high-energy sandy or bioturbated sites mediate fermentation via the Embden–Meyerhof–Parnas pathway, in a manner uncoupled from anaerobic respiration. Prolonged anoxic incubation suggests that this uncoupling lasts up to 160 h. In contrast, microbial communities in anoxic muddy sediments (smaller median grain size) generally completely oxidized ^13^C glucose to ^13^CO_2_, consistent with the classical redox cascade model. We also unexpectedly observed that fermentation occurred under oxic conditions in permeable sediments. These observations were further confirmed using pure cultures of four bacteria isolated from permeable sediments. Our results suggest that microbial communities adapted to variable oxygen regimes metabolize glucose (and likely other organic molecules) through fermentation uncoupled to respiration during transient anoxic conditions.

## Introduction

**Figure 1:**
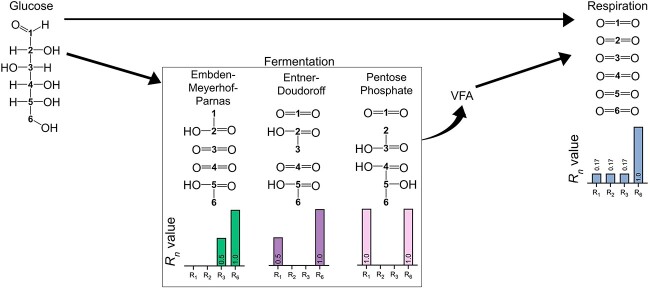
Different carbon degradation pathways from the six-carbon glucose molecule; glucose can be fermented into: acetate and CO_2_ by the EMP pathway; CO_2_, acetate, and lactate by the PP pathway; or CO_2_ and acetate by the ED pathway; the oxidation of VFAs produced during fermentation (acetate, lactate, etc.) is typically coupled to the reduction of anoxic terminal electron acceptors; respiration can also occur directly using the glucose with no prior fermentation; *R_n_* values of each pathway, derived from ^13^CO_2_ ratios of different glucose isotopologues (see Calculations), are represented underneath; if fermentation remains uncoupled from respiration, VFAs are not consumed and *R_n_* values remain that of whichever fermentation occurs.

In the absence of oxygen, microbial oxidation of organic matter is initiated by hydrolysis of macromolecules into smaller constituents (e.g. sugars, fatty acids, amino acids), which are then fermented to dissolved inorganic carbon (DIC), molecular hydrogen (H_2_), alcohols, and volatile fatty acids (VFAs). Heterotrophic bacteria largely use one of three fermentation pathways for degradation of glucose: the Embden–Meyerhof–Parnas (EMP), pentose phosphate (PP) or Entner–Doudoroff (ED) pathways (Fig. 1) [1]. EMP fermentation is generally most widespread and active [1], with recent studies indicating at least 90% of obligate and facultative anaerobes use the EMP pathway [2]. After fermentation, the reduced compounds produced (VFAs, alcohols, and H_2_) are rapidly oxidized by respiring bacteria, i.e. terminal respiration coupled with fermentation (Fig. 1). This paradigm has been relatively well developed and studied in cohesive sediments (i.e. muds and silts), which typically have stable physical and redox regimes that allow a close coupling between fermenting and respiring bacteria, e.g. Schulz and Zabel [3].

In contrast to cohesive sediments, microorganisms in permeable and bioturbated sediments experience dynamic redox regimes. In permeable sediments (i.e. sands and gravels), wave oscillations, sediment movement, and currents drive advective pore water exchange, which can lead to shifts between oxic and anoxic conditions on timescales of minutes to hours [4, 5]. In bioturbated sediments, similar shifts between oxic and anoxic conditions have been observed in relation to faunal pumping [6]. These short-term redox variations select for metabolically flexible microbes, adapted to varying electron donor and acceptor availability. Indeed, the dominant bacteria in permeable sediments are facultative anaerobic bacteria from the families *Flavobacteriaceae* and *Woeseiaceae*, which are capable of aerobic respiration, anaerobic respiration, and fermentation [7-9]. In turn, these communities appear to oxidize organic carbon under anoxic conditions through distinct pathways to those in cohesive sediments. Specifically, they mediate organic carbon fermentation, but do not fully reoxidize derived end-products through respiration. Evidence for this comes from lower-than-expected accumulation of end-products of anoxic respiration such as nitrogen (N_2_), iron (Fe^2+^), and sulfide (H_2_S) compared to DIC production, accompanied by the accumulation of hydrogen observed in flow-through reactors in anoxic permeable sediments [9, 10]. However, to date all inferences of fermentation have been indirect, and it remains unresolved through what pathways organic carbon is oxidized in anoxic permeable or bioturbated sediments.

Here we used a differentially labeled ^13^C glucose assay to compare oxidation pathways in sandy and muddy sediments. Nine sandy and three muddy sediments from Australia and Denmark were incubated with position-specific ^13^C-labeled glucose isotopologues. Labeled either on the first carbon (1-^13^C), second carbon (2-^13^C), third carbon (3-^13^C), or all six carbons (^13^C_6_) (Fig. 1), the ratio of ^13^CO_2_ produced in each treatment (*R_n_*) was then used to determine the dominant carbon oxidation metabolism. Although this broad approach has long been used by biochemists for determining fermentation pathways of cultivated microbes [11, 12], this is the first study to apply this approach quantitatively and to a range of sediments with complex microbial communities for direct comparison. We hypothesized that microbial respiration would dominate over fermentation of glucose in cohesive sediments, compared to permeable sediments, except if heavily bioturbated. Moreover, we expected permeable sediments to show increased coupling of fermentation to respiration after prolonged anoxic incubations of days to weeks in line with previously observed shifts in microbial communities under extended anoxia [9]. To support these inferences, we also extended these assays to four bacterial isolates from permeable sediments, including members of the dominant family *Flavobacteriaceae*.

## Materials and methods

### Study sites

Between March 2019 and March 2021, nine sandy sediments and three muddy sediments were sampled at sites across Australia and Denmark (Fig. S1). Sites spanned temperate to tropical locations and included both silicate and carbonate sediments. The biogeochemistry of each site has been previously studied, and references to these studies are provided in Table S1. Sandy sites were selected to encompass a range of hydrodynamic regimes and included three high-energy surface sands and three low-energy surface sands. Sites were grouped into high or low energy based on the observation of colored depth profiles of sediment cores and wave height and frequency. Sites with greater wave action displayed yellow oxidized sediments at greater depths (~15 cm deep) with anoxic grey layers below this. At lower energy sites, the anoxic sediment (5–10 cm deep) was much darker. Higher-energy locations included Werribee Beach and Melbourne Beach, both enclosed within a large bay, yet still frequently exposed to medium to large waves. Heron Reef was also deemed high energy, located on a coral cay in the Great Barrier Reef. Sites deemed lower energy included Melbourne Harbour, located further up the beach from Melbourne Beach and partially shielded from oncoming waves by a harbor break wall. Low-energy sediments were also collected in Denmark from Fællesstrand, a shallow marine lagoon on the northeast coast of Fyn, and Hjerting Beach, which is shielded from open ocean by a barrier spit complex known as Skallingen. In addition, deeper lower energy sediments were collected at Melbourne Beach, Melbourne Harbour, and Fællesstrand. Both surface (0–15 cm) and deep (15–30 cm) sediments were collected from the subtidal zone. Surficial muddy sediments were sampled from estuaries around Victoria (Yarra, Gippsland, Patterson). Patterson sediment was heavily infaunated, which is typical for this site [13], while no significant fauna was observed at other sites.

### Slurry incubations

Sandy sediments were sieved (2-mm mesh) to remove large debris and fauna, and seawater was filtered (1.6–11 μm) to remove most pelagic species potentially present in overlying water. Muddy sediments remained unsieved. Slurries were prepared with 20 g wet sediment and 35 ml filtered seawater in 60 ml incubation vials before sealing with a butyl rubber stopper (Sigma-Aldrich) and Wheaton closed-top seals (Sigma-Aldrich). Anoxic treatments were purged with nitrogen gas to exclude O_2_, while the headspace of the oxic treatment remained as unamended air, corresponding to a dissolved oxygen concentration of ~230 μM. Melbourne Beach and Melbourne Harbour deep sediments (15–30 cm) were prepared in an N_2_-filled anoxic chamber to avoid exposure to oxygen. Fællesstrand deep sediments were not prepared in an anoxic chamber and hence were momentarily exposed to oxygen during set up. After a designated preincubation time (explained below), ^13^C-labeled glucose isotopologues (1-^13^C, 2-^13^C, 3-^13^C, or ^13^C_6_, Cambridge Isotope Laboratories Inc., 98%–99%) were added (50 μM) in parallel with three replicate slurries each (Fig. S2) and placed on the orbital shaker (150 rpm, dark, 20°C) for 4 h. Subsequently, these slurries were left to settle for 5 min, then opened and subsampled.

To determine organic carbon oxidation pathways at four distinct time points, we employed preincubation periods of 0, 1, and 7 days before addition of ^13^C-labeled glucose followed by a 4-h incubation (Fig. S2). These preincubation periods were chosen because previous studies have shown that sulfide production in permeable sediment commences after several days of anoxia and that there is a shift in the microbial community to sulfate reducers over this period [9]. We therefore expected to see a distinct temporal sequence from fermentation to increased respiration over this timeframe. For the 0-day preincubation, glucose was added immediately after the preparation of the slurries. For 1- and 7-day preincubations, slurries were put on an orbital shaker for gentle agitation (150 rpm) in a light-proof box at 20°C for the designated period before glucose addition. After the preincubation period, the ^13^C-labeled glucose isotopologues were added, while three initial slurries (t_0_) were opened and subsampled simultaneously, as described below, to determine the background concentrations of DIC and ^13^CO_2_ (Fig. S2). Slurries with added ^13^C-labeled glucose were then replaced on the orbital shaker. Note that experiments were not set up to mirror the constantly fluctuating redox conditions; instead prolonged oxic and anoxic incubations enabled observation of otherwise transient processes.

### Flow through reactor experiment

Surface sand (0–10 cm) from Melbourne Beach was sieved (1 mm mesh), homogenised, and packed into 6 cylindrical reactors (4.2 cm length, 4.8 cm inner diameter) as previously described[9, 10, 14]. Filtered seawater (0.7 μm, GF/F) amended with 1-^13^C- or ^13^C_6_-labeled glucose (50 μM) was pumped through flow through reactors (FTRs) using a peristaltic pump at a flow rate of ~45 ml h^−1^ with residence time of the pore water ~1 h. Both labeled seawater reservoirs were contained in 10-L high-density polyethylene carboys, which were replaced every ~48 h. Due to the large volume of the reservoirs, 1-^13^C and ^13^C_6_ labeled glucose were most cost-effective so were selected over other ^13^C glucose isotopologues. O_2_ levels were monitored from the FTR outlet using a flow-through O_2_-sensitive probe (PyroScience FireSting). Glass syringes collected seawater from the outlets and reservoirs for DIC, ^13^CO_2_, and VFA analysis every few hours. Both seawater reservoirs were bubbled with ambient air at the first sampling point (0 h), before transitioning to anoxia by purging with 800 ppm CO_2_ in pure N_2_ using a digital gas mixer (Vögtlin).

### Isotopic and biogeochemical measurements

Samples for measurement of DIC concentration (3 ml) and ^13^CO_2_ concentration (12 ml) were collected in gastight glass vials (Labco Exetainer), preserved with 10 μl 6% HgCl_2_ and stored with no headspace. Prior to ^13^CO_2_ analysis, 4 ml of sample in the 12-ml vial was replaced with helium. Phosphoric acid (12.5 mM) was added to the sample to convert DIC to CO_2_ before being analyzed on a Hydra 20–22 Continuous Flow Isotope Ratio Mass Spectrometer (CF-IRMS; Sercon Ltd., UK). Total DIC concentration was measured using a DIC analyser (Apollo SciTech). Nitrate and sulfate concentrations were not measured; however, we know from previous measurements background that nitrate is likely to be <10 μM [10], and sulfate will be in the order of seawater concentrations at 28 mM. Sediment grains were sized using a Beckham Coulter LP13 320 particle sizer after soaking in sodium hexametaphosphate for 24 h and sonicating for 10 min. FTR samples for VFA analysis (2 ml) were collected in 4 ml ashed borosilicate glass vials with Teflon lined lids (Sigma Aldrich) and frozen. Upon analysis, samples were thawed and derivatized as previously described (Albert and Martens, 1997), before injection into reverse phase High Performance Liquid Chromatography (HPLC) combined with preconcentrator and guard column (Agilent SB-C8 4.6 × 12.5 mm) and analytical column (Agilent SB-C8 4.6 × 250 mm).

### Isolation, cultivation, and sequencing of bacteria

We designed a strategy to culture facultative anaerobes representative of the permeable marine sediment communities of Port Philip Bay, Victoria. Sediment and seawater from Melbourne Beach were collected in November 2020 and combined in 180 ml vials to form a slurry. The slurry was supplemented with 1 mM glucose and sealed with a butyl rubber stopper before being made anoxic by purging with N_2_. The vial was then placed on an orbital shaker (150 rpm, dark) at room temperature (20°C). After a 14-day incubation, plates of Marine Agar 2216 medium (Difco) supplemented with 1 mM glucose were prepared in Petri dishes and inoculated with a portion of the slurry and incubated aerobically at 30°C for 3 days. Following this incubation, individual bacterial colonies were repeatedly transferred to fresh Marine Agar 2216 + 1-mM glucose plates for purification and identification. 16S rRNA genes from each bacteria were amplified using colony PCR and visualized via gel electrophoresis [15], before extraction (Isolate II PCR & Gel Kit, Bioline) and whole-genome sequencing. Cellular DNA was sent to MHTP Medical Genomics Facility, Hudson Institute of Medical Research for whole-genome sequencing. The library preparation was performed using the Illumina Nextera XT DNA library prep kit with unique dual Indexing, which was then passed to 150 bp paired end sequencing on a NextSeq2000 platform (Illumina). Raw shotgun sequences were subjected to quality filtering using the BBDuk function of the BBTools v38.80 (https://sourceforge.net/projects/bbmap/), which sequentially removed contaminating adapters (k-mer size of 23 and hamming distance of 1), PhiX sequences (k-mer size of 31 and hamming distance of 1), bases from 3′ ends with a Phred score below 20, and resultant reads with lengths shorter than 50 bp. Quality-filtered reads were assembled using Unicycler v.0.4.7 (—mode normal, —keep 0) to obtain draft genomes [16]. The purity of the isolates was corroborated by the absence of foreign DNA and the presence of a sole 16S rRNA gene in the assembled genomes. The taxonomy of the newly isolated bacteria was assigned by GTDB-Tk v1.4.0 [17] with reference to the Genome Taxonomy Database (GTDB) R06-RS202 [18]. Annotation of the genomic features and metabolic capabilities was performed using DRAM v1.2.4 [19], with dbCAN2 database, MEROPS peptidase database, and KEGG protein database (accessed 22 November 2021). We additionally searched the genomes against our custom databases (doi: 10.26180/c.5230745) for the presence of key metabolic marker genes involved in using various electron acceptors and donors using DIAMOND v.2.0.11 [20], with cut-offs reported previously [21].

### Pure culture ^13^C isotopologue glucose assay and volatile fatty acid measurements

Marine Broth 2216 medium (Difco) was prepared in 180-ml vials, sealed with butyl rubber stoppers, and the headspace kept under anoxic (purged with N_2_) and oxic (purged with air) conditions. Each vial was then inoculated with a bacterial strain to an OD_600_ of 0.02 before addition of 50 μM ^13^C-labeled glucose isotopologues (1-^13^C, 2-^13^C, 3-^13^C, or ^13^C_6_) as outlined previously. Simultaneously, three initial incubations (t_0_) that were not treated with glucose were opened and subsampled to determine background concentrations of ^13^CO_2_. Vials with added ^13^C-labeled glucose were then placed on an orbital shaker (150 rpm, 30°C) for 4 h before final sampling of ^13^CO_2_. Samples for measurement of ^13^CO_2_ concentration were collected and analyzed as described in Isotopic and biogeochemical measurements section.

### Bacterial incubations for volatile fatty acid analysis

Incubations were set up to monitor growth (OD_600_) of both bacteria under oxic and anoxic conditions as well as VFA production. About 50 ml Marine Broth 2216 medium (Difco) supplemented with 1 mM glucose was prepared in 180-ml vials before bacteria was inoculated to an OD_600_ of 0.05 and vials sealed with butyl rubber stoppers. Incubations were made anoxic by purging with N_2_ or constantly flushed with ambient air to ensure the headspace remained oxic. Vials were then placed on an orbital shaker (150 rpm) at 30°C for a week. About 1 ml sample was removed each day and the optical density at 600 nm (OD_600_) measured (1-cm cuvette; Eppendorf BioSpectrometer basic). Based on the anoxic growth curves of each bacteria, these cultures were incubated for 2 and 4 days, which correlates to early and mid-stationary phase. At these time points, the culture was transferred to centrifuge tubes and centrifuged at 4500 × *g*. Samples of the supernatant (3 ml) were filtered (0.2 μm) and frozen in 4-ml ashed borosilicate glass vials with Teflon lined lids (Sigma-Aldrich) for VFA analysis. Media controls without bacteria were also run. Analysis of VFA samples proceeded as described in Isotopic and biogeochemical measurements section.

### Calculations

#### Slurry incubations

Depending on the ^13^C-labeled glucose isotopologue added (1-^13^C, 2-^13^C, 3-^13^C, ^13^C_6_) and the metabolism taking place, carbon in different positions will be converted to ^13^CO_2_, and a ^13^CO_2_ ratio (*R_n_*) can be calculated (Fig. 1).

The excess concentration of ^13^CO_2_ produced in incubations amended with glucose labeled at the 1C, 2C, and 3C positions was derived using the difference in *r* from the beginning (t_0_) to the end of the 4-h incubations and DIC concentration such that


(1) \begin{equation*} {\left[^{13} CO_2\right]}_{n-^{13}C}=\Delta r\ \left[ DIC\right] \end{equation*}

where *r* is the ratio of masses 45/44 and *n* = 1, 2, 3 (position of labeled carbon atom).

The same is calculated for the excess concentration of ^13^CO_2_ produced in incubations amended with glucose labeled on all six carbon atoms.


(2) \begin{equation*} {\left[^{13} CO_2\right]}_{{13_C}_6}=\Delta r\ \left[ DIC\right] \end{equation*}

^13^CO_2_ ratios (*R_n_*) for each labeled carbon position are then derived by normalizing against the ^13^C_6_ treatment (Fig. S3, Table S2), such that


(3) \begin{equation*} {R}_n=\frac{{\left[^{13} CO_2\right]}_{n-^{13}C}}{{\left[^{13} CO_2\right]}_{{13_C}_6}} \end{equation*}

where *n* = 1, 2, 3.

and


(4) \begin{equation*} {R}_6=\frac{{\left[^{13} CO_2\right]}_{{13_C}_6}}{{\left[^{13} CO_2\right]}_{{13_C}_6}}=1 \end{equation*}

For example, respiration of 3-^13^C glucose results in a ^13^CO_2_ ratio of 0.17, as only one out of six carbons becomes ^13^CO_2_ (Fig. 1).


(5) \begin{equation*} {R}_3=\frac{{\left[^{13} CO_2\right]}_{3-^{13}C}}{{\left[^{13} CO_2\right]}_{{13_C}_6}}=\frac{1}{6}=0.17 \end{equation*}

When respiration is inactive, *R_n_* will resemble that of a fermentation pathway (EMP, PP, ED).

For example, when bacteria undergo EMP fermentation using 3-^13^C glucose, one of two CO_2_ produced will be labeled, such that


(6) \begin{equation*} {R}_3=\frac{{\left[^{13} CO_2\right]}_{3-^{13}C}}{{\left[^{13} CO_2\right]}_{{13_C}_6}}=\frac{1}{2}=0.5 \end{equation*}

Alternatively, if bacteria undergo PP fermentation using 1-^13^C glucose, the only CO_2_ produced will be labeled, such that


(7) \begin{equation*} {R}_1=\frac{{\left[^{13} CO_2\right]}_{1-^{13}C}}{{\left[^{13} CO_2\right]}_{{13_C}_6}}=\frac{1}{1}=1 \end{equation*}

Total carbon oxidation was then distributed into fractions as respiration-driven (${f}_{resp}$), EMP fermentation-driven $({f}_{EMP\ ferm}$), ED fermentation-driven $({f}_{ED\ ferm}$), or PP fermentation-driven $({f}_{PP\ ferm}$).


(8) \begin{equation*} {f}_{resp}+{f}_{EMP\ ferm}+{f}_{ED\ ferm}+{f}_{PP\ ferm}=1 \end{equation*}

**Figure 2:**
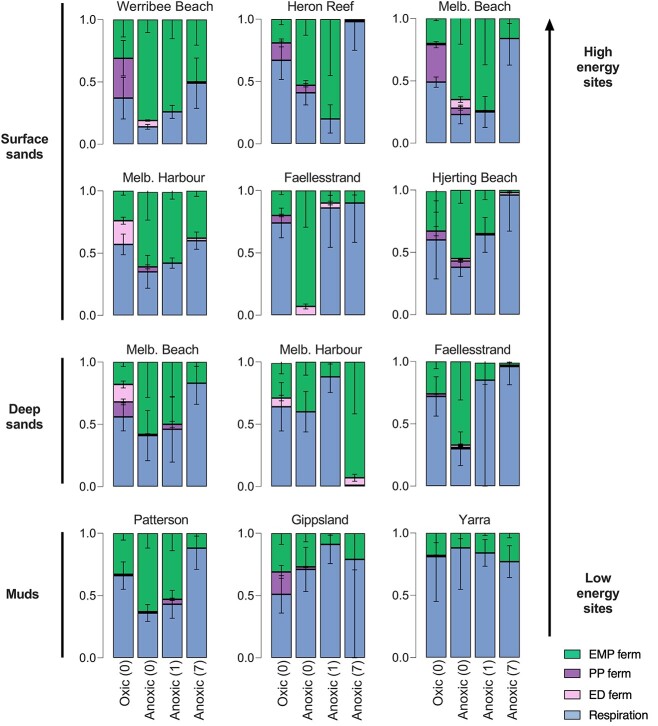
Fractions of respiration, ED, EM, and PP fermentation occurring at each site during each incubation, as determined by R package Rsolnp using ^13^CO_2_ ratios (see Calculations); incubations include oxic (0), anoxic (0), anoxic (1), and anoxic (7); numbers in parentheses represent number of days of anoxia pretreatment before the glucose assay (4-h duration) was undertaken; sands and muds are arranged from high-energy sites to low-energy sites and are also grouped into surface and deep.

A best fit for the contribution of the metabolisms (respiration, EMP fermentation, ED fermentation, PP fermentation) to the total rate of CO_2_ production was estimated from all *R_n_* values (Fig. 2 and Table S3). This fit was performed by minimizing the total sum of squares of the error between observed and theoretical *R_n_* values using the R package Rsolnp [22] and subject to the constraints that all contributions are positive and add up to 1 [23]. Carbon oxidation for each incubation is often distributed as more than one fraction and, in these instances, we defined the dominant metabolic pathway as that responsible for the largest fraction of ^13^CO_2_ production. It should be noted that some fermentations do not produce CO_2_ [24], and therefore, this approach will not quantify these pathways. However, we observed less than or equal to the expected accumulation of VFAs (see results) based on the calculated fractions of fermentation, and it is therefore unlikely such pathways are occurring at significant rates.

#### Bacterial cultures

^13^CO_2_ ratios of pure culture glucose assays were calculated similarly to above (Fig. S4, Table S2); however, DIC was not accounted for. Instead, the excess concentration of ^13^CO_2_ produced in incubations amended with glucose isotopologues (1-^13^C, 2-^13^C, 3-^13^C, ^13^C_6_) was derived using only the difference in *r* from the beginning (t_0_) to the end of the 4-h incubations such that


(9) \begin{equation*} {\left[^{13} CO_2\right]}_{n-^{13}C}=\Delta r \end{equation*}

where *r* is the ratio of masses 45/44 and *n* = 1, 2, 3 (position of labeled carbon atom).

The same is calculated for the excess concentration of ^13^CO_2_ produced in incubations amended with glucose labeled on all six carbon atoms.


(10) \begin{equation*} {\left[^{13} CO_2\right]}_{{13_C}_6}=\Delta r \end{equation*}

^13^CO_2_ ratios (*R_n_*) for each labeled carbon position and a best fit for the fractional contribution of the four metabolisms are then derived as above (Equations (3)–(8)). The standard deviation of each metabolism is determined by propagating the standard deviations of the raw ratios through the calculation.

#### Flow through reactor experiment

The excess concentration of ^13^CO_2_ produced in the reactor effluent amended with glucose labeled at the 1st and on all six carbon atoms is also calculated using Equations (1) and (2) except the difference in *r* is determined between the reservoir and sediment-packed reactors. As only 1-^13^C and ^13^C_6_ labeled glucose were used, *R_1_* is derived using Equations (3) and (4) (Tables S2 and S5). Given that we only used two isotopologues for this experiment, we assumed that respiration and EMP fermentation dominated (consistent with the four isotopologue experiment), and total carbon oxidation was distributed as respiration-driven (${f}_{resp}$) or fermentation-driven $({f}_{EMP\ ferm}$) based on the prominence of *R_1_* values:


(11) \begin{equation*} {f}_{EMP\ ferm}+{f}_{resp}=1 \end{equation*}

(12) \begin{equation*} {R}_1=\frac{1}{6}\ {f}_{resp}+0\ {f}_{EMP\ ferm} \end{equation*}

where *R_1_* is determined following Equation (3) and 1/6 and 0 follow from the theoretical *R_n_* values (see Fig. 1).


(13) \begin{equation*} {f}_{EMP\ ferm}=1-6R_1\end{equation*}

Solving Equations (11) and (12) gives Equation (13), which predicts the proportion of carbon oxidation driven by EMP fermentation at each time point. Following Equation (11), the remaining carbon oxidation is then assumed to be the fraction driven by respiration $({f}_{resp}$). Again, EMP fermentation or respiration is then determined as the dominant metabolic pathway when the fraction is >0.5.

## Results

### Fermentation dominates anoxic glucose degradation in permeable sediments

We distinguished glucose oxidation of each sediment as respiration-driven (${f}_{resp}$), EMP fermentation-driven $({f}_{EMP\ ferm}$), ED fermentation-driven $({f}_{ED\ ferm}$), or PP fermentation-driven $({f}_{PP\ ferm}$) through the pathways summarized in Fig. 1 (see “Calculations”). Across all sediments, glucose metabolism was typically dominated by either respiration or EMP fermentation, though ED fermentation did contribute in some incubations (Figs 2 and S3, Tables S2 and S3). In line with the classical redox cascade, respiration is dominant in low-energy, unperturbated muddy sediments (Yarra, Gippsland) across both oxic and anoxic incubations up until 7 days.

In contrast, fermentation was the dominant glucose mineralization pathway in most permeable sediments. Microbial communities in surface sands from multiple sites all undertook EMP fermentation under anoxia. EMP fermentation typically dominated $({f}_{EMP\ ferm}$ > 0.5) within the first hours of anoxia (Anoxic 0) for nearly all surface sediments and persisted for up to 24 h (Anoxic 1). Respiration typically increased following 3 and 7 days of anoxic incubations. While some of the deep sediments show fractions of EMP fermentation under initial anoxia, they are all more respiration-driven than the surface sands of the same location. The observations that respiration is higher in deeper sands or following long-term incubations are consistent with more stable redox conditions favoring activity of sulfate-reducing bacteria. Surprisingly, substantial fractions of both EMP and ED fermentation were also observed in oxic incubations, particularly in high-energy surface sands including Werribee and Melbourne Beach.

**Figure 3:**
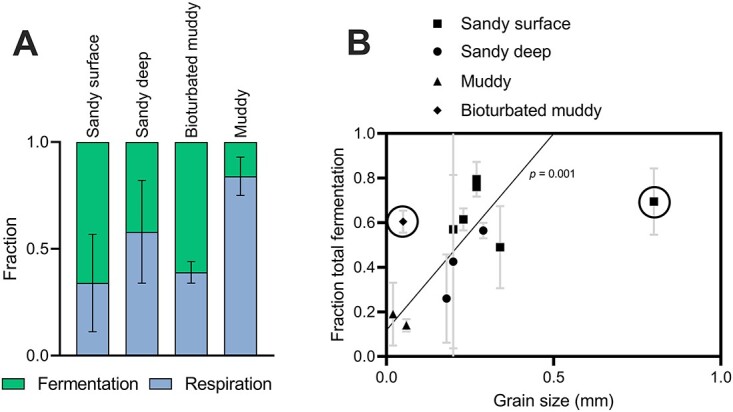
(A) Fractions of total fermentation$({f}_{EMP\ ferm}+{f}_{ED\ ferm}+{f}_{PP\ ferm}$) and respiration (${f}_{resp}$) grouped into sediment types and averaged over the first day of anoxia (anoxic 0–1). Sediment types include surface sands (*n* = 6; Werribee Beach, Heron Reef, Melbourne Beach, Melbourne Harbour, Fællesstrand, Hjerting Beach), deep sands (*n* = 3; Melbourne Beach, Melbourne Harbour, Fællesstrand), muds (*n* = 2; Gippsland, Yarra), and bioturbated mud (*n* = 1; Patterson) (see Table S1); error bars depict the standard deviation. (B) The average fraction of total fermentation$({f}_{EMP\ ferm}+{f}_{ED\ ferm}+{f}_{PP\ ferm}$) over the first day of anoxia (Anoxic 0–1) against median grain size at each site; Heron Reef and Patterson mud are omitted as outliers (circled), while grain sizing data for Fællesstrand deep is absent; line of best fit has equation *y* = 1.76× + 0.118 (*R*^2^ = 0.60, *P* = .001); different sediments are represented by different symbols as follows: surface sands (■), deep sands (●), muds (▲), and bioturbated mud (♦); error bars depict the range.

Comparing the total fraction of fermentation $({f}_{EMP\ ferm}$ + ${f}_{ED\ ferm}$ + ${f}_{PP\ ferm}$) with respiration (${f}_{resp}$) shows surface and deep sands have similar average fractions under initial anoxia, though large differences are seen in muds (Fig. 3A). The total fraction of fermentation on the first day of anoxia (Anoxic 0–1) for each sediment was then compared against median grain size as a measure of hydrodynamic energy (Fig. 3B). High-energy sites with larger grain sizes such as Werribee Beach had the highest fraction of fermentation, while fine-grained muddy sediments such as Yarra Estuary had fermentation fractions close to 0. Altogether, this produced a strong positive correlation (*R*^2^ = 0.60, *P* = .001) when the notable outliers of highly bioturbated muds (Patterson) and large grain size carbonate sands (Heron) are excluded (Fig. 3B). Average total fractions of fermentation of each sediment were also plotted against DIC production, but no correlation was observed (data not shown).

### A shift from fermentation to respiration during long-term anoxic incubations

**Figure 4:**
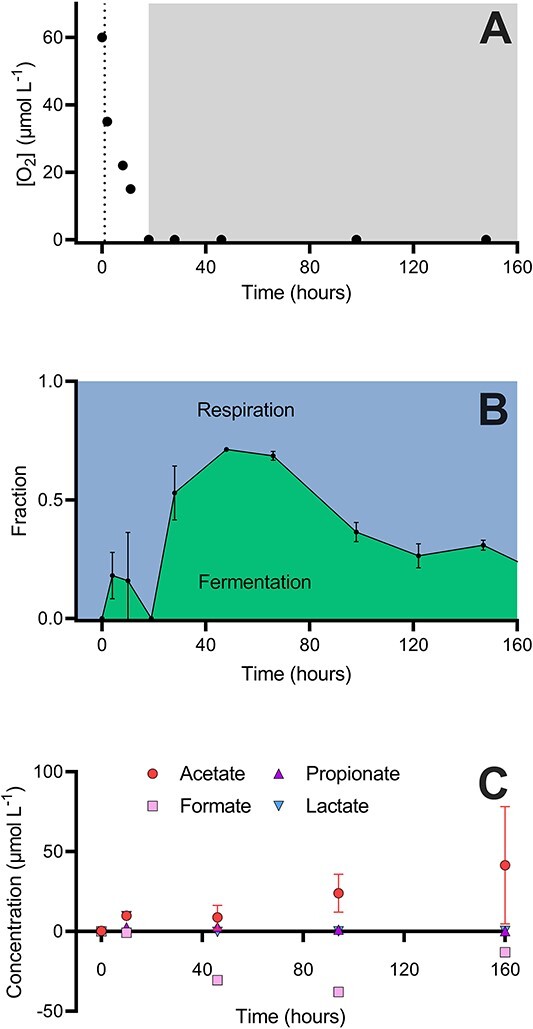
FTR experiments were run under oxic conditions for 18 h then transitioned to anoxia (dotted line, A) and remained anoxic for the rest of the experiment (grey shading); fractions of EMP fermentation $({f}_{EMP\ ferm}$) and respiration (${f}_{resp}$) were estimated from *R_1_* values (B, see calculations); respiration is dark grey and EMP fermentation is light grey; net concentration changes (reservoir values subtracted from FTR outlets) of acetate (●), formate (■), propionate (▲) and lactate (▼, C); acetate and formate concentration apply to the left y-axis while propionate and lactate values are on the right y-axis; error bars show the standard deviation.

We investigated the effect of extended anoxic conditions on the transition between fermentation and respiration using FTR experiments where we measured fermentation, respiration, and VFA production (Fig. 4). This experiment showed the dominance of respiration during the initial oxic conditions from 0 to 18 h (Fig. 4A and B, Tables S4 and S5), then transitioned to a dominance of EMP fermentation at 46 h (after 28 h anoxia). This was followed by a return to a dominance of respiration at 100 h where it remained until the experiment finished at 160 h. We measured the concentrations of various VFAs to determine whether fermentative end products were excreted (Fig. 4C, Table S7). At the onset of anoxia, acetate and lactate production dominated (10 μM mean concentration for both), after which acetate production dominated reaching a maximum concentration of 40 μM at 160 h. Formate was consistently consumed by the FTRs.

In order to account for fermentation products, we undertook a mass balance of CO_2_ and acetate produced in the reservoir and FTR at 46 h (anoxic fermenting) and 160 h (anoxic respiring, Table 1). At 160 h, we were able to account for 102% of the glucose respired as acetate and CO_2_; however, at 46 h, we were only able to account for 66% of the glucose in the form of CO_2_ and acetate. To compare the observed accumulation of acetate with that expected from fermentation, we applied the fraction of fermentation from the ^13^C glucose assay to the measured amount of ^13^CO_2_ produced and calculated an expected acetate accumulation equivalent. At 160 h, we were able to account for 110% of the expected acetate accumulation, but we were only able to account for 12% of the expected acetate at 46 h indicating an unknown sink for fermentation products.

**Table 1:** The amount of CO_2_ and acetate accumulated in the reservoir and FTR in μM C equivalents as per Fig. 5; CO_2_ accumulation in the reservoir was based on acetate accumulation assuming a 2:1 acetate:CO_2_ C equivalent for fermentation C_6_H_12_O_6_ + 2 H_2_O ➔ 2 CH_3_COOH + 2 CO_2_ + 4 H_2_; acetate accumulation in the FTR was based on the difference between acetate concentrations in the reservoir and FTR outlet; CO_2_ production in the FTR was based on measured ^13^CO_2_ production in the FTRs. ${f}_{EMP\ ferm}$ is calculated as per Equation (9); calculated acetate production in the FTR is based on ^13^CO_2_ production × ${f}_{EMP\ ferm}$ × 2 (acetate CO_2_ stoichiometry as per above).

	Time point	
	46 h	160 h
Acetate reservoir (μM C equiv)	52	32
CO_2_ reservoir (μM C equiv)	26	16
Acetate FTR (μM C equiv)	18	82
^13^CO_2_ FTR (μM C equiv)	102	176
**Sum** (μM C equiv)	**198**	**306**
% C recovered compared to glucose added	66	102
${f}_{EMP\ ferm}$	0.71	0.21
Calc acetate production FTR (μM C equiv)	145	74
% Acetate FTR of calc acetate production	12	111

### Bacterial cultures validate occurrence of oxic and anoxic fermentation

**Figure 5:**
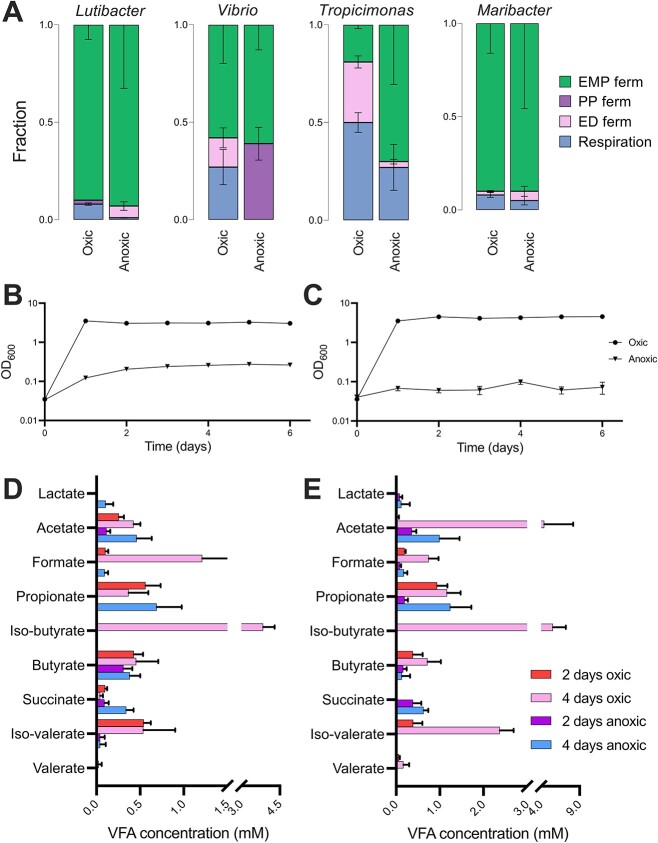
A fractions of respiration, ED, EM, and PP fermentation occurring in oxic and anoxic incubations of bacterial species *Lutibacter* sp*.*, *Vibrio* sp., *Tropicimonas* sp*.*, and *Maribacter* sp*.* as determined by R package Rsolnp using ^13^CO_2_ ratios (see Calculations); growth curves of B *Lutibacter* and C *Maribacter* over 1-week incubation under both oxic and anoxic conditions; VFA concentrations in the cultures of D *Lutibacter* and E *Maribacter* after 2 and 4 days under oxic and anoxic conditions; error bars show the standard deviation.

To further compare respiration, fermentation, and VFA production dynamics in bacterial cultures under oxic and anoxic conditions, we isolated species *Lutibacter* sp., *Vibrio* sp., *Tropicimonas* sp., and *Maribacter* sp. from marine sediments and quantified the fractions of fermentation and respiration under short-term oxic and anoxic conditions (Figs 5A and S4, Tables S2 and S6). All species showed a dominance of EMP fermentation $({f}_{EMP\ ferm}$ > 0.5) under anoxic conditions, though we also observed a distinct fraction of PP fermentation in *Vibrio*$({f}_{PP\ ferm}$ = 0.39). Under oxic conditions, metabolism was surprisingly also dominated by fermentation through the EMP pathway for most species $({f}_{EMP\ ferm}$ > 0.5), with the exception of *Tropicimonas* for which respiration comprised half of CO_2_ production (${f}_{resp}$ = 0.50) (Fig. 5A). *Tropicimonas* and *Maribacter*, both capable of respiration under anoxic conditions, possess genes for nitrous oxide reduction (Fig. S5). Additionally, *Tropicimonas,* which displayed the greatest fraction of anoxic respiration, has genes for nitrate and nitric oxide reduction (Fig. S5). It should be noted that the different pathways are integrated over the 4-h experiment and that it is likely that they occur sequentially as the glucose is consumed and thermodynamics change. In extended incubations to measure VFA accumulation, both *Lutibacter* and *Maribacter* reached stationary phase under oxic conditions within 1 day of incubation (Fig. 5B and C). *Lutibacter* was able to grow under anoxic conditions, albeit at a much slower rate and to a lower yield than under oxic conditions, reaching stationary phase after ~3 days. *Maribacter* showed no growth under anoxic conditions. Under oxic conditions, both bacteria produced VFAs including acetate, formate, propionate, isobutyrate, butyrate, succinate, isovalerate, and valerate (Fig. 5D and E), with concentrations highest after 4 days under oxic conditions in the *Maribacter* culture. Under anoxic conditions, the highest concentrations of VFAs were observed after 4 days of anoxia, including acetate, propionate butyrate, and succinate.

## Discussion

Most carbohydrates within the sediment exist in a polymeric form, which are hydrolyzed to monomeric sugars such as glucose. Our addition of 50 μM glucose was most likely higher than natural concentrations of ~1 μM in permeable sediments [25]. Of critical importance here is the concentration of glucose relative to the saturating concentration for glucose uptake (fermentation), which can range from 0.2 to 23 μM for particle-associated bacteria [26]. At the lower end of this range, our glucose addition is unlikely to have increased the rate of glucose uptake, while at the higher range, this could have greatly stimulated glucose uptake (fermentation) leading to an accumulation of fermentation products before respiring bacteria were able to assimilate them [27]. It is therefore possible that the “fermentation” proxy we are measuring with the isotopologues represents a temporal decoupling between fermenting and respiring bacteria. If this were occurring, then we would expect to see a substantial release of VFAs after glucose addition, which was not observed (Table 1, Fig. 4C see also discussion below). It should therefore be recognized that fermentation (or some fraction of it) may have been induced by the glucose tracer addition.

Fermentation of glucose dominated in most permeable sediments, with EMP fermentation being the predominant carbon oxidation pathway $({f}_{EMP\ ferm}$ > 0.5, Figs 2 and 4B). Within permeable sediments, it is likely that redox conditions are constantly fluctuating due to periodic incorporation of oxygen and other electron acceptors into the sands by sediment resuspension and advective pore water flow [5, 28]. The microbial community in these high-energy sands is known to be dominated by metabolically flexible generalist bacteria of families *Flavobacteriaceae* and *Woeseiaceae* that ferment under anoxic conditions [7-9, 29], and the culture experiments confirmed this with all species showing a dominance of fermentation under anoxic conditions (Fig. 5A). Metabolism in these high-energy sandy sediments trended toward respiration between 1 to 7 days of anoxia (Fig. 2). In agreement, FTR incubations show the evolution of fermentation in Melbourne Beach sediments, with fermentation dominating upon initial anoxia before switching back to respiration after ~2 days of anoxia (Fig. 4A and B). This is consistent with previous observations that after 4–10 days anoxia, there is an enrichment of sulfate reducers of families *Desulfobacteraceae* and *Desulfobulbaceae*, which leads to a recoupling between fermentative and anaerobic respiratory bacteria [7-9, 30].

Comparing the fractions of fermentation within the first day of anoxia (Anoxic 0–1) against median grain size for each site showed a positive correlation (*P* = 0.001), with two notable outliers (Fig. 3B). Hydrodynamic energy at each site controls both grain size and the frequency of sediment re-oxygenation [28, 31]. Coarse-grained sediments will be more oxygenated and likely dominated by facultative aerobes, and finer grain sizes more anoxic and dominated by coupled anaerobic fermentative and respiratory bacteria. This relationship is therefore consistent with our hypothesis that fermentation uncoupled to respiration will dominate in more dynamic higher-energy settings.

The two noted outliers that do not conform to this relationship are a highly bioturbated mud and coarse grain carbonate sediments. In the case of the bioturbated mud, there was much more fermentation than would be expected based on the grain size alone and fermentation dominated immediately after the onset of anoxia (Fig. 2). The exact mechanism driving the fermentation is unclear, but we suggest it could be linked to the activity of benthic fauna that can oxygenate sediments on a time scale comparable to that of advective transport [32-35]. Similar to sandy sediments, pore waters fluctuate between oxic and anoxic conditions, which may select for metabolically versatile bacteria that can survive in both oxic and anoxic conditions [7-9]. Previous studies of infaunated sediments have shown that microbial communities indeed differ between bioturbated and nonbioturbated zones [36, 37], with dynamic and re-worked bioturbated sediments featuring generalist fermenters such as *Flavobacteriaceae* [37]*.* The occurrence of fermentation at the Patterson River suggests that our hypothesis of flexible generalist communities adapting to dynamic conditions by fermenting extends beyond sandy sediments to other temporally oxic/anoxic sediments. In the case of the coarse-grained carbonate sediments, there was much more respiration than would be expected from grain size. Carbonate sediment grains are known to be porous and highly biologically active and hence harbor permanently anoxic zones within the grains, which can enhance anoxic processes such as denitrification [38]. As such, it is highly likely that even though this sediment type is highly oxygenated around the grains, there is a community of coupled fermentative and respiring bacteria within the grains that fully respire the added glucose tracer.

As noted previously, the 50 μM glucose addition may have stimulated fermentation and hence led to a temporal decoupling of respiration and fermentation. If this is the case here, then an alternative explanation for our observations is that cohesive and less disturbed sediments have a higher capacity to assimilate fermentation products or a lower saturation concentration for glucose fermentation. Further studies with direct glucose measurements and lower glucose tracer additions are required to definitively validate this hypothesis.

The observation that significant rates of fermentation occurred in the high-energy sediments under oxic conditions (Fig. 2) was unexpected. These observations were consistent with the results from the pure culture experiments, which showed that facultatively aerobic bacteria isolated from Melbourne Beach including *Lutibacter, Vibrio*, and *Maribacter* undertook fermentation $({f}_{EMP\ ferm}$ > 0.5) during oxic conditions (Fig. 5A). *Vibrio* and *Tropicimonas* undertook significant fractions of ED fermentation as well $({f}_{ED\ ferm}$ = 0.15 and 0.31, respectively). The production of VFAs under oxic conditions in both pure culture experiments is also consistent with oxic fermentation. Fermentation under oxic conditions at high glucose concentrations (>10 mM range) has been well documented in the literature and is known as the “Crabtree” effect or overflow metabolism in bacteria [39-41]. Although fermentation yields less energy per mol of glucose than oxidative phosphorylation (complete oxidation to CO_2_), it can proceed much more rapidly and therefore yield more energy [42] as well as maximizing growth relative to proteome formation [43]. Within the context of cancer cells, this metabolism is known as the Warburg effect, and it has been argued the reason for this is that it rebalances the Krebs cycle in proliferating cells to provide anabolic (biomolecules) as opposed to catabolic (CO_2_, ATP) products required for cell growth [44]. Fermentation under oxic conditions may therefore convey an ecological advantage in dynamic environments such as permeable sediments supporting rapid growth when oxygen and organic matter is often transiently available [45]. The fact that we were able to observe fermentation under oxic conditions in both cultures and natural sediments exposed to lower glucose concentrations (50 μM for the isotope assays) suggests that oxic fermentation may be a common phenomenon in bacteria even at low glucose concentrations and warrants further investigation. In addition, the increased predominance of the ED fermentation pathway under oxic conditions at some sites and cultures (e.g. *Maribacter* and *Tropicimonas*) is consistent with the paradigm that it can be a major catabolic pathway for glucose under oxic conditions [46].

Our results also support previous observations that there is very little acetate production relative to CO_2_ in anoxic permeable sediments [10]. A mass balance for CO_2_ and acetate produced in the FTR at 46 and 160 h provides further insight into the fate of glucose under anoxic fermenting (46 h) and respiring (160 h) conditions. At 160 h, we were able to account for 102% of the glucose respired as acetate and CO_2_ (Table 1). However, at 46 h, we were only able to account for 66% of the glucose in the form of CO_2_ and acetate. Furthermore, if we apply the fraction of fermentation calculated using the ^13^C glucose assay to the measured amount of ^13^CO_2_ produced, we can also calculate an expected acetate accumulation equivalent (Table 1). At 160 h, the calculated acetate accumulation was 74 μM C equivalents, compared to 82 μM C equivalents measured (110% of that expected). At 46 h, the calculated acetate accumulation was 145 μM C equivalents compared to a measured value of 18 μM C equivalents (12% of that expected). This suggests that the fermentation uncoupled to respiration observed here is not as a result of a transient accumulation of VFAs stimulated by glucose addition [27, 47]. It is possible that the missing acetate was assimilated by polyhydroxyalkanoate-accumulating organisms, which have been observed in permeable sediments [48] or stored as lipids [10, 30]. Consistent with this, the culture experiments showed that *Lutibacter* and *Maribacter* produced negligible VFAs after 2 days anoxia. Furthermore, *Lutibacter* was able to increase its biomass under anoxic conditions, which suggests *Lutibacter* may be able to assimilate some of the fermentation products into storage molecules, which have recently been suggested to comprise an under-appreciated form of biomass [49]. The exact nature of this process remains to be determined and requires further investigation.

## Conclusion

Using isotopologues of ^13^C labeled glucose, we show through slurry and FTR incubations that EMP fermentation occurs in transiently anoxic sandy sediments, remaining uncoupled from respiration processes for up to 160 h. Factors controlling the frequency of sediment re-oxygenation such as hydrodynamic energy and bioturbation exert a strong control on the extent to which glucose fermentation decouples from respiration at the onset of anoxia. Oxic fermentation was observed in both sands and bacterial cultures and suggests that this process may be significant in the environment. Our understanding of the extent to which this occurs with naturally present carbohydrates and the fate of the organic fermentation products remains incomplete and the question remains as to what the final products of fermentation are.

## Supplementary Material

supplementary_material_wrae001

## Data Availability

The sequences of the four assembled genomes were deposited to the NCBI Sequence Read Archive under the BioProject accession number PRJNA609151.

## References

[1] Romano A , Conway T. Evolution of carbohydrate metabolic pathways. Res Microbiol 1996;147:448–55. 10.1016/0923-2508(96)83998-2. 9084754

[2] Flamholz A , Noor E, Bar-Even A et al. Glycolytic strategy as a tradeoff between energy yield and protein cost. Proc Natl Acad Sci U S A 2013;110:10039–44. 10.1073/pnas.1215283110. 23630264 PMC3683749

[3] Schulz HD , Zabel M. Marine Geochemistry. Springer, Berlin, 2005.

[4] McGinnis DF , Sommer S, Lorke A et al. Quantifying tidally driven benthic oxygen exchange across permeable sediments: an aquatic eddy correlation study. J Geophys Res 2014;119:6918–32.

[5] Precht E , Franke U, Polerecky L et al. Oxygen dynamics in permeable sediments with wave-driven pore water exchange. Limnol Oceanogr 2004;49:693–705. 10.4319/lo.2004.49.3.0693.

[6] Wenzhöfer F , Glud RN. Small-scale spatial and temporal variability in coastal benthic O2 dynamics: effects of fauna activity. Limnol Oceanogr 2004;49:1471–81. 10.4319/lo.2004.49.5.1471.

[7] Chen Y-J , Leung PM, Wood JL et al. Metabolic flexibility allows bacterial habitat generalists to become dominant in a frequently disturbed ecosystem. ISME J 2021;15:2986–3004. 33941890 10.1038/s41396-021-00988-w PMC8443593

[8] Chen Y-J , Leung PM, Cook PLM et al. Hydrodynamic disturbance controls microbial community assembly and biogeochemical processes in coastal sediments. ISME J 2022;16:750–63. 10.1038/s41396-021-01111-9. 34584214 PMC8857189

[9] Kessler AJ , Chen Y-J, Waite DW et al. Bacterial fermentation and respiration processes are uncoupled in anoxic permeable sediments. Nat Microbiol 2019;4:1014–23. 10.1038/s41564-019-0391-z. 30858573

[10] Bourke MF , Marriott PJ, Glud RN et al. Metabolism in anoxic permeable sediments is dominated by eukaryotic dark fermentation. Nat Geosci 2017;10:30–5. 10.1038/ngeo2843. 28070216 PMC5217482

[11] De Bruyn IN , Holzapfel WH, Visser L et al. Glucose metabolism by *Lactobacillus divergens*. Microbiology 1988;134:2103–9. 10.1099/00221287-134-8-2103. 3253406

[12] Schäfer T , Xavier KB, Santos H et al. Glucose fermentation to acetate and alanine in resting cell suspensions of *Pyrococcus furiosus*: proposal of a novel glycolytic pathway based on 13C labelling data and enzyme activities. FEMS Microbiol Lett 1994;121:107–14. 10.1111/j.1574-6968.1994.tb07083.x.

[13] Kessler AJ , Roberts KL, Bissett A et al. Biogeochemical controls on the relative importance of denitrification and dissimilatory nitrate reduction to ammonium in estuaries. Glob Biogeochem Cycles 2018;32:1045–57. 10.1029/2018GB005908.

[14] Evrard V , Glud RN, Cook PL. The kinetics of denitrification in permeable sediments. Biogeochemistry 2013;113:563–72. 10.1007/s10533-012-9789-x.

[15] Bergkessel M , Guthrie C. Chapter twenty five-colony PCR. In: Lorsch J. (ed.), Methods in Enzymology. Academic Press, San Diego, 2013, 299–309. 10.1016/B978-0-12-418687-3.00025-2 24011056

[16] Wick RR , Judd LM, Gorrie CL et al. Unicycler: resolving bacterial genome assemblies from short and long sequencing reads. PLOS Compl Biol 2017;13:e1005595. 10.1371/journal.pcbi.1005595. PMC5481147 28594827

[17] Chaumeil PA , Aaron JM, Hugenholtz P et al. GTDB-Tk: a toolkit to classify genomes with the genome taxonomy database. Bioinform 2020;36:1925–7. 10.1093/bioinformatics/btz848 PMC7703759 31730192

[18] Parks DH , Chuvochina M, Rinke C et al. GTDB: an ongoing census of bacterial and archaeal diversity through a phylogenetically consistent, rank normalized and complete genome-based taxonomy. Nucleic Acids Res 2022;50:D785–94. 10.1093/nar/gkab776. 34520557 PMC8728215

[19] Shaffer M , Borton MA, McGivern BB et al. DRAM for distilling microbial metabolism to automate the curation of microbiome function. Nucleic Acids Res 2020;48:8883–900. 10.1093/nar/gkaa621. 32766782 PMC7498326

[20] Buchfink B , Xie C, Huson DH. Fast and sensitive protein alignment using DIAMOND. Nat Methods 2015;12:59–60. 10.1038/nmeth.3176. 25402007

[21] Ortiz M , Leung PM, Shelley G et al. Multiple energy sources and metabolic strategies sustain microbial diversity in Antarctic desert soils. Proc Natl Acad Sci U S A 2021;118:e2025322118. 10.1073/pnas.2025322118. 34732568 PMC8609440

[22] Ghalanos A , Theussl S. Rsolnp: general non-linear optimization using augmented Lagrange multiplier method. R package version 116 edn 2015.

[23] Ye Y . Interior Algorithms for Linear, Quadratic, and Linearly Constrained Non-Linear Programming. PhD Thesis,. Stanford University, Stanford, 1987.

[24] Ciani M , Comitini F, Mannazzu I. Fermentation. In: Jørgensen SE, Fath BD (eds), Encyclopedia of Ecology. Oxford: Academic Press, 2008, 1548–57, 10.1016/B978-008045405-4.00272-X.

[25] Meyer-Reil LA . Uptake of glucose by bacteria in the sediment. Mar Biol 1978;44:293–8. 10.1007/BF00390892.

[26] Ayo B , Unanue M, Azúa I et al. Kinetics of glucose and amino acid uptake by attached and free-living marine bacteria in oligotrophic waters. Mar Biol 2001;138:1071–6. 10.1007/s002270000518.

[27] Arnosti C , Finke N, Larsen O et al. Anoxic carbon degradation in Arctic sediments: microbial transformations of complex substrates. Geochim Cosmochim Acta 2005;69:2309–20. 10.1016/j.gca.2004.11.011.

[28] Precht E , Huettel M. Rapid wave-driven advective pore water exchange in a permeable coastal sediment. J Sea Res 2004;51:93–107. 10.1016/j.seares.2003.07.003.

[29] Mußmann M , Pjevac P, Krüger K et al. Genomic repertoire of the Woeseiaceae/JTB255, cosmopolitan and abundant core members of microbial communities in marine sediments. ISME J 2017;11:1276–81. 10.1038/ismej.2016.185. 28060363 PMC5437919

[30] Kessler AJ , Rogers A, Cyronak T et al. Pore water conditions driving calcium carbonate dissolution in reef sands. Geochim Cosmochim Acta 2020;279:16–28. 10.1016/j.gca.2020.04.001.

[31] Huettel M , Ziebis W, Forster S et al. Advective transport affecting metal and nutrient distributions and interfacial fluxes in permeable sediments. Geochim Cosmochim Acta 1998;62:613–31. 10.1016/S0016-7037(97)00371-2.

[32] Aller RC . The effects of macrobenthos on chemical properties of marine sediment and overlying water. In: McCall PL, Tevesz MJS. (eds). Animal-Sediment Relations. Springer, New York, 1982, 53–102.

[33] Huettel M , Røy H, Precht E et al. Hydrodynamical impact on biogeochemical processes in aquatic sediments. In: Kronvang B, Faganeli J, Ogrinc N. (eds). The Interactions between Sediments and Water. Springer, Dordrecht, 2003, 231–6.

[34] Kristensen E . Organic matter diagenesis at the oxic/anoxic interface in coastal marine sediments, with emphasis on the role of burrowing animals. In: Liebezeit G, Dittman S, Kroencke I. (eds). Life at Interfaces and under Extreme Conditions. Springer, Dordrecht, 2000, 1–24.

[35] Santos IR , Eyre BD, Huettel M. The driving forces of porewater and groundwater flow in permeable coastal sediments: a review. Estuar Coast Shelf Sci 2012;98:1–15. 10.1016/j.ecss.2011.10.024.

[36] Chen X , Andersen TJ, Morono Y et al. Bioturbation as a key driver behind the dominance of bacteria over archaea in near-surface sediment. Sci Rep 2017;7:1–14. 28546547 10.1038/s41598-017-02295-x PMC5445093

[37] Deng L , Bölsterli D, Kristensen E et al. Macrofaunal control of microbial community structure in continental margin sediments. Proc Natl Acad Sci U S A 2020;117:15911–22. 10.1073/pnas.1917494117. 32576690 PMC7376573

[38] Kessler AJ , Cardenas MB, Santos IR et al. Enhancement of denitrification in permeable carbonate sediment due to intra-granular porosity: a multi-scale modelling analysis. Geochim Cosmochim Acta 2014;141:440–53. 10.1016/j.gca.2014.06.028.

[39] Lara AR , Taymaz-Nikerel H, Mashego MR et al. Fast dynamic response of the fermentative metabolism of *Escherichia coli* to aerobic and anaerobic glucose pulses. Biotechnol Bioeng 2009;104:1153–61. 10.1002/bit.22503. 19685524

[40] Sun JL , Zhang SK, Chen JY et al. Metabolic profiling of Staphylococcus aureus cultivated under aerobic and anaerobic conditions with (1) H NMR-based nontargeted analysis. Can J Microbiol 2012;58:709–18. 10.1139/w2012-046. 22571732

[41] Thierie J , Penninckx M. Crabtree effect. In: Flickinger MC. (ed). Encyclopedia of Industrial Biotechnology: Bioprocess, Bioseparation and Cell Technology. Wiley, Hoboken, 2010, 1774–90.

[42] Swain A , Fagan W, F. A mathematical model of the Warburg effect: effects of cell size, shape and substrate availability on growth and metabolism in bacteria. Math Biosci Eng 2019;16:168–86. 10.3934/mbe.2019009. 30674115

[43] Basan M , Hui S, Okano H et al. Overflow metabolism in *Escherichia coli* results from efficient proteome allocation. Nature 2015;528:99–104. 10.1038/nature15765. 26632588 PMC4843128

[44] Vander Heiden MG , Cantley LC, Thompson CB. Understanding the Warburg effect: the metabolic requirements of cell proliferation. Science 2009;324:1029–33. 10.1126/science.1160809. 19460998 PMC2849637

[45] Huettel M , Cook PLM, Janssen F et al. Transport and degradation of a dinoflagellate algal bloom in a permeable sandy sediment. Mar Ecol Prog Ser 2007;340:139–53. 10.3354/meps340139.

[46] Spector MP . Metabolism, central (intermediary). In: Schaechter M (ed). Encyclopedia of Microbiology3rd edn. Oxford: Academic Press, 2009, 242–64, 10.1016/B978-012373944-5.00078-X.

[47] Finke N , Jorgensen BB. Response of fermentation and sulfate reduction to experimental temperature changes in temperate and Arctic marine sediments. ISME J 2008;2:815–29. 10.1038/ismej.2008.20. 18309360

[48] Dyksma S , Lenk S, Sawicka JE et al. Uncultured gammaproteobacteria and desulfobacteraceae account for major acetate assimilation in a coastal marine sediment. Front Microbiol 2018;9:3124. 10.3389/fmicb.2018.03124. 30619197 PMC6305295

[49] Mason-Jones K , Breidenbach A, Dyckmans J et al. Intracellular carbon storage by microorganisms is an overlooked pathway of biomass growth. Nat Commun 2023;14:2240. 10.1038/s41467-023-37713-4. 37076457 PMC10115882

