# Supplementary material for: Microorganisms oxidize glucose through distinct pathways in permeable and cohesive sediments
Source: ISME J. 2024 Jan 30;18(1):wrae001. doi: 10.1093/ismejo/wrae001 (PMC10939381; doi:10.1093/ismejo/wrae001)
Supplement: supplementary_material_wrae001 [file supplementary_material_wrae001.zip › Supplementary Information.docx]

**Supplementary Information**


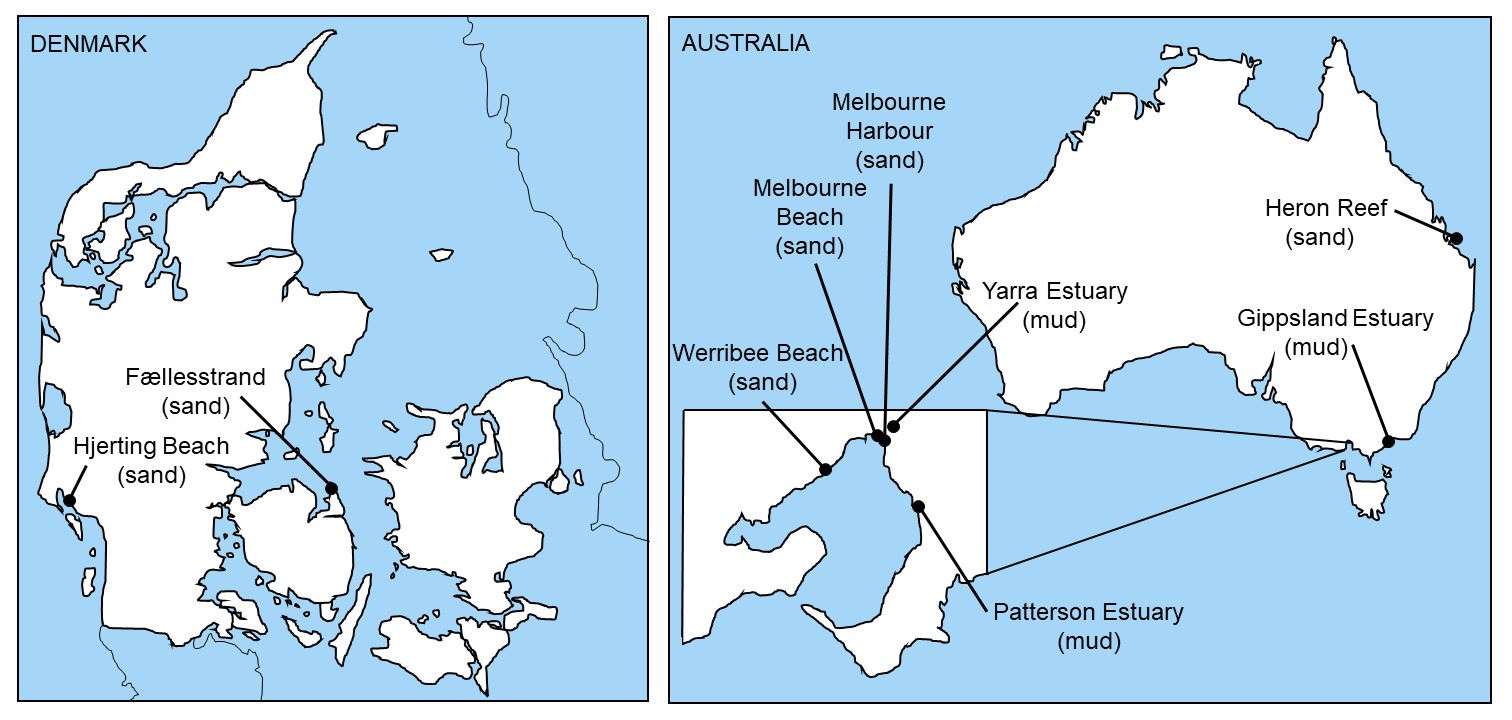


**Supplementary Figure S1.** Locations of field sites sampled in Denmark and Australia.


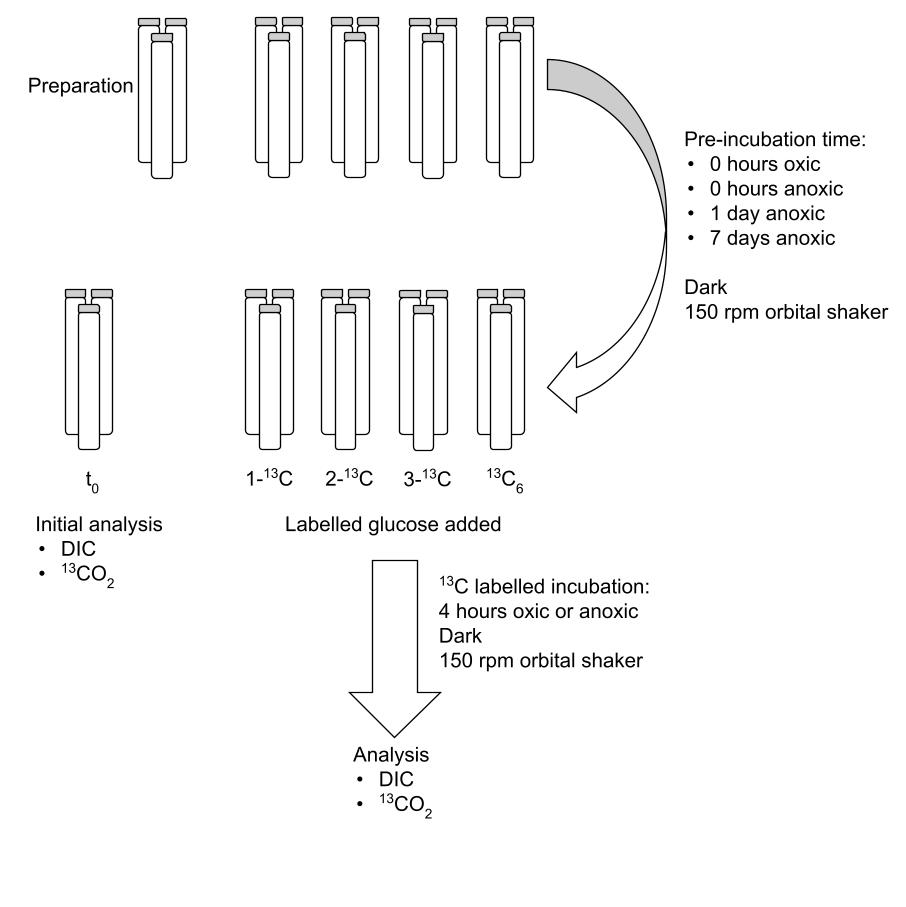


**Supplementary Figure S2.** Schematic diagram of methods. Slurries are prepared and then incubated in the dark at 150 rpm for differing pre-incubation periods, under oxic or anoxic conditions. After either 0, 1 or 7 days, labelled ^13^C glucose isotopologues are added to the vials (1-^13^C, 2-^13^C, 3-^13^C, or ^13^C_6_), or used for initial analysis (t_0_). Of those with labelled ^13^C glucose added, another incubation begins on the shaker table (150 rpm) under the same conditions (dark, oxic/anoxic). After 4 hours, samples are collected for analysis.

**
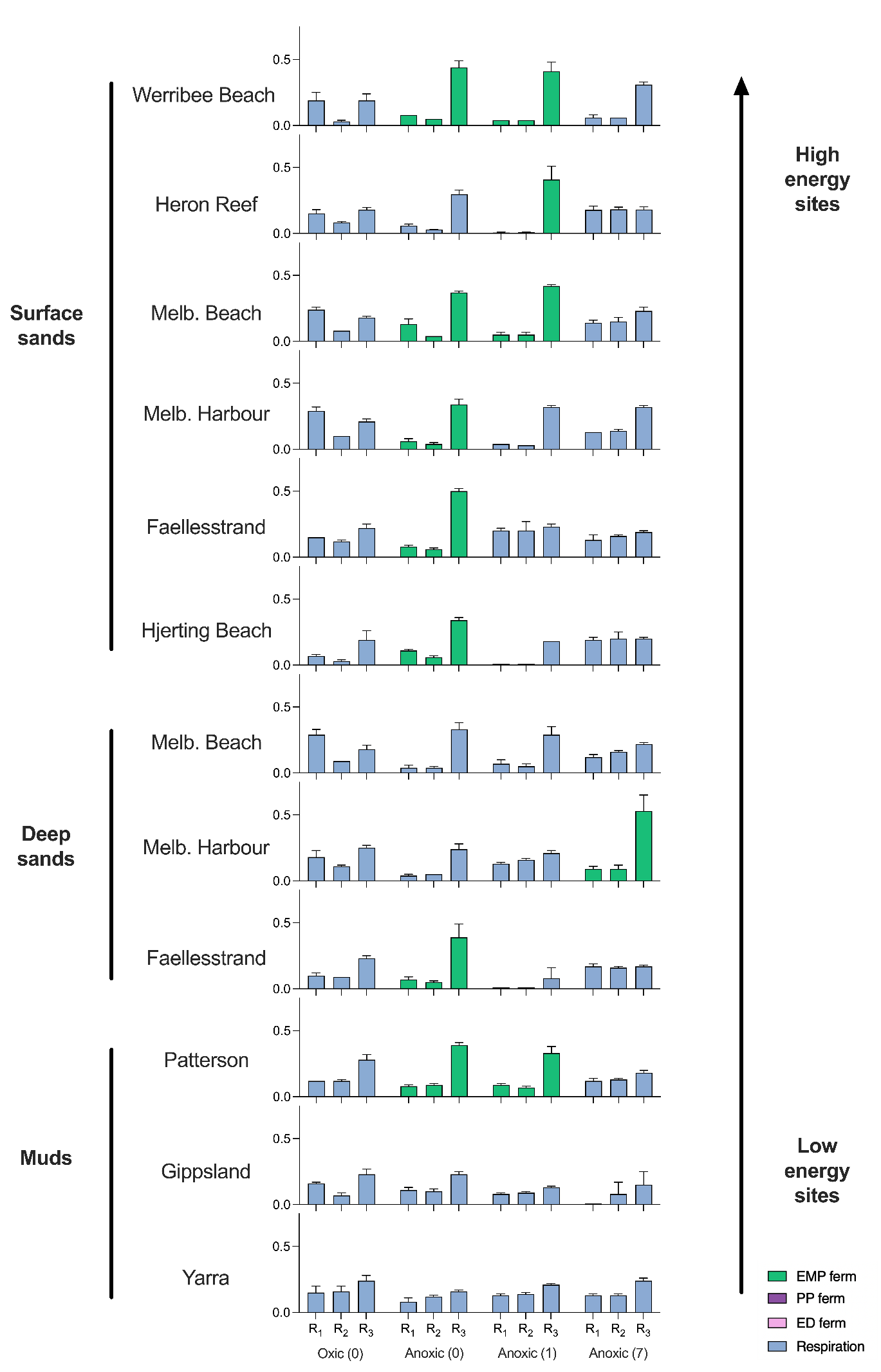
**

**Supplementary Figure S3** R_n_ values of each incubation at all sites sampled in Australia and Denmark. R_1_, R_2_ and R_3_ values are derived from ^13^CO_2_ ratios during incubations with isotopologues labeled on the 1^st^, 2^nd^ and 3^rd^ carbon positions (see **Calculations, Fig S2**). Incubations include Oxic (0), Anoxic (0), Anoxic (1) and Anoxic (7) with numbers in parentheses representing number of days of anoxia before the glucose assay was undertaken. Sands and muds are arranged from high energy sites to low energy sites and are also grouped into surface and deep. Incubations are coloured based on their greatest metabolic fraction: respiration ($f_{resp}$), EMP fermentation ${(f}_{EMP ferm}$), ED fermentation ${(f}_{ED ferm}$) or PP fermentation ${(f}_{PP ferm}$). Error bars depict the standard deviation.

**
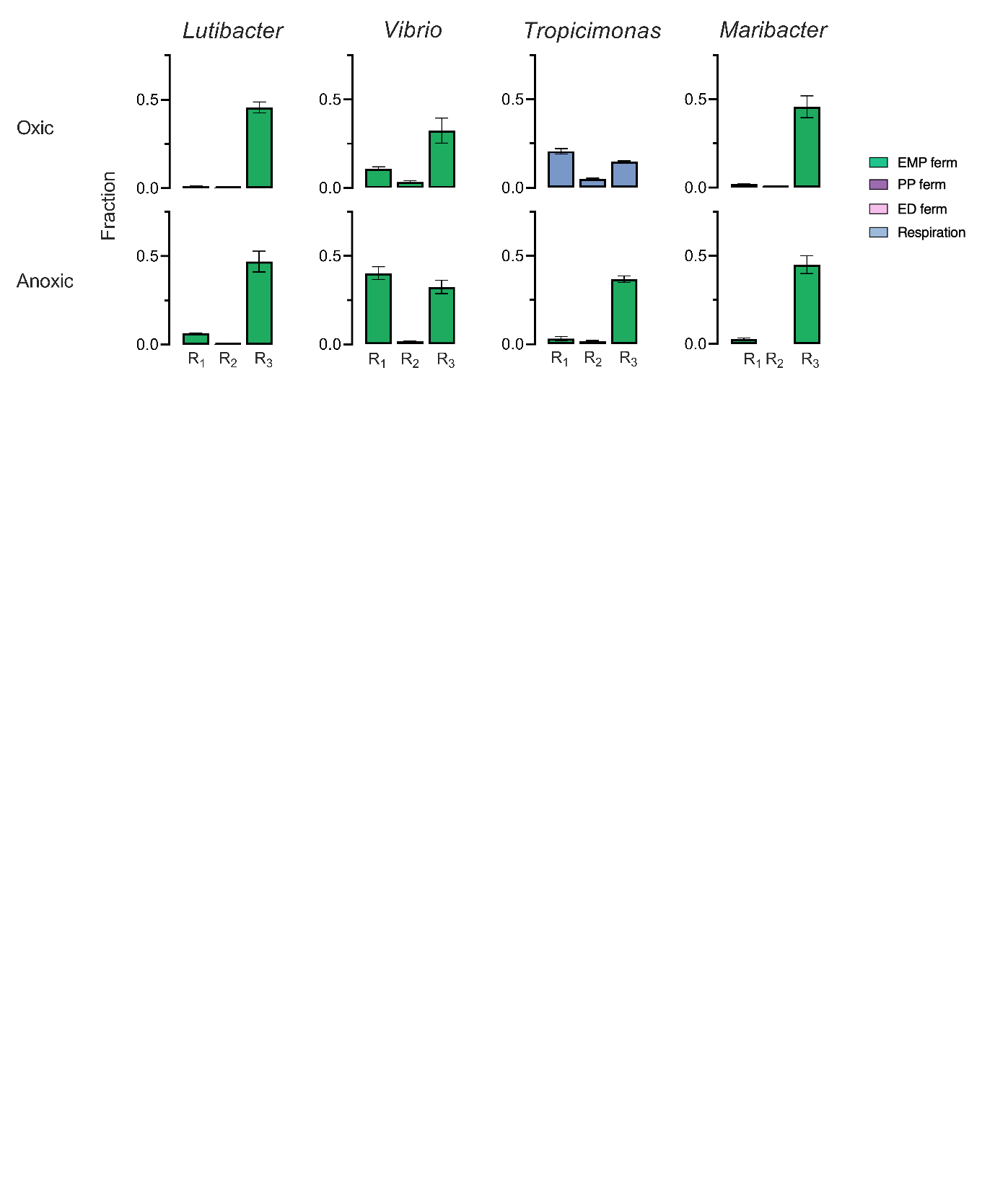
**

**Supplementary Figure S4** R_n_ values of oxic and anoxic incubations of bacterial species Lutibacter sp., Vibrio sp., Tropicimonas sp., and Maribacter sp. where R_1_, R_2_ and R_3_ values are derived from ^13^CO_2_ ratios during incubations with isotopologues labeled on the 1^st^, 2^nd^ and 3^rd^ carbon positions (see **Calculations, Fig S2**). Incubations are coloured depending on the dominant metabolic pathway (see **Fig S3**), defined when the fraction > 0.5 (see **Calculations**). Error bars depict the standard deviation.


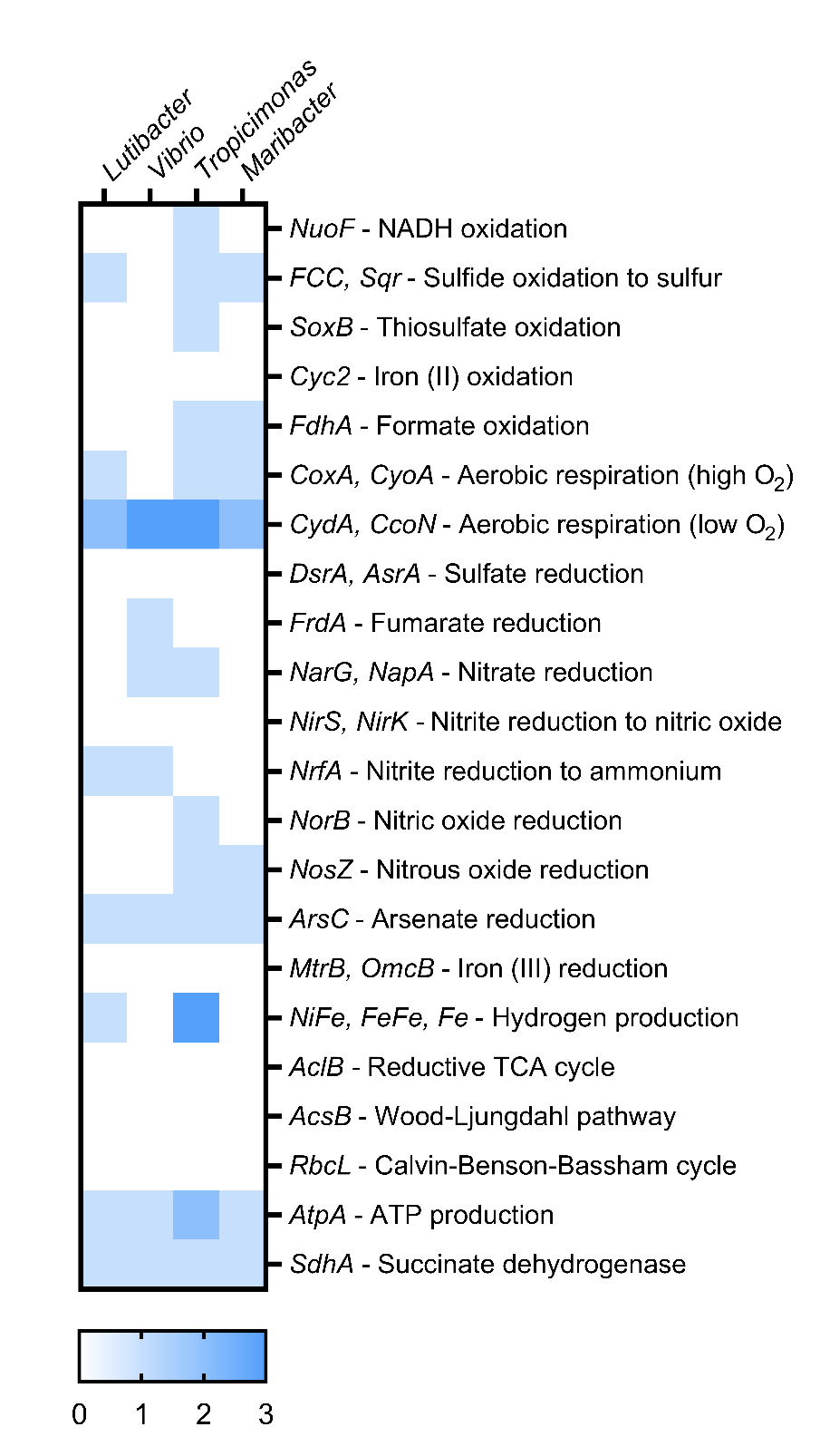


**Supplementary Figure S5** Homology-based searches detected key metabolic genes including those for oxidation of electron donors, reduction of electron acceptors and fixation of inorganic carbon in the genomes of *Lutibacter, Vibrio, Tropicimonas* and *Maribacter.*

**Supplementary Table S1.** Field sites visited with type of sediment, location of site, sediment layers sampled, site description, incubation times, products measured and dates of collection.

| **Sediment type** | **Location** | **Sediment layer** | **Site description (redox status)** | **Country** | **Incubation times** | **Products analysed** | **Date** |
| --- | --- | --- | --- | --- | --- | --- | --- |
| Sandy  Silicate | Werribee Beach (Wong et al 2013, Wong et al 2014) (37.970723°S, 144.703538°E) | Surface | High energy site  No sulfidic zone observed | Australia | Oxic (0)  Anoxic (0, 1, 7) | ^13^CO_2_, DIC | 12/3/2019 |
| Sandy  Carbonate | Heron Reef (Santos et al 2011) (23.443360°S, 151.912121°E) | Surface | High energy site  No sulfidic zone observed | Australia | Oxic (0)  Anoxic (0, 1, 7) | ^13^CO_2_, DIC | 1/11/2018 |
| Sandy  Silicate | Melbourne Beach (Bourke et al 2017, Chen et al 2021, Chen et al 2022, Kessler et al 2019, Santos et al 2011) (37.856283°S, 144.964258°E) | Surface  Deep | High energy site  Sulfidic zone from 15 cm deep | Australia | Oxic (0)  Anoxic (0, 1, 7)  FTR | ^13^CO_2_, DIC, VFA | 17/4/2019  3/4/2019  27/3/2021 |
| Sandy  Silicate | Melbourne Harbour (Chen et al 2021) (37.861310°S, 144.969734°E) | Surface  Deep | Low energy site  Very dark sulfidic zone from 10 cm deep | Australia | Oxic (0)  Anoxic (0, 1, 7) | ^13^CO_2_, DIC | 24/3/2019  18/3/2019 |
| Sandy  Silicate | Hjerting Beach  (55.520611°S, 8.356180°E) | Surface | Low energy site  Very dark sulfidic zone from 10 cm deep | Denmark | Oxic (0)  Anoxic (0, 1, 7) | ^13^CO_2_, DIC | 10/9/2019 |
| Sandy  Silicate | Fӕllesstrand Beach (Kristensen 1993, Kristensen and Hansen 1995) (55.610013°S, 10.612165°E) | Surface  Deep | Bioturbated  Low energy site  Sulfidic zone from 15 cm deep | Denmark | Oxic (0)  Anoxic (0, 1, 7) | ^13^CO_2_, DIC | 3/9/2019 |
| Muddy | Yarra Estuary (Scotch College)  (Roberts et al 2012, Roberts et al 2014) (37.833572°S, 145.022625°E) | Surface | Very sulfidic  Non-bioturbated | Australia | Oxic (0)  Anoxic (0, 1, 7) | ^13^CO_2_, DIC | 1/4/2019 |
| Muddy | Gippsland Estuary (Lake King) (37.894161°S, 147.727627°E) | Surface | Very sulfidic  Non-bioturbated | Australia | Oxic (0)  Anoxic (0, 1, 7) | ^13^CO_2_, DIC | 16/2/2020 |
| Muddy | Patterson Estuary (Cook et al 2007) (38.070394°S, 145.128078°E) | Surface | Heavily bioturbated | Australia | Oxic (0)  Anoxic (0, 1, 7) | ^13^CO_2_, DIC | 5/6/2019 |

**Supplementary Table S2 (xlsx).** Raw data including Ratio 45/44, DIC concentrations and *R_n_* values for each slurry, FTR and pure culture. Outliers were excluded as determined by a Q test at 95% confidence level.

**Supplementary Table S3.** The relative contribution of respiration, ED fermentation, EMP fermentation and PP fermentation to each incubation as determined using the Rsolnp R package. DIC produced (µmol/L) over the 7 day period for each site.

| **Site** | **Pre-incubation** | **Resp.** | **ED ferm.** | **EMP ferm.** | **PP ferm.** | **DIC produced (µmol/L)** |
| --- | --- | --- | --- | --- | --- | --- |
| Werribee Beach (surface) | Oxic (0) | 0.37 | 0.32 | 0.31 | 0 |  |
|  | Anoxic (0) | 0.14 | 0 | 0.81 | 0.05 |  |
|  | Anoxic (1) | 0.26 | 0 | 0.74 | 0 |  |
|  | Anoxic (7) | 0.49 | 0.01 | 0.50 | 0 | 1547 ± 191 |
| Heron Reef (surface) | Oxic (0) | 0.67 | 0.14 | 0.19 | 0 |  |
|  | Anoxic (0) | 0.41 | 0.06 | 0.53 | 0 |  |
|  | Anoxic (1) | 0.20 | 0 | 0.80 | 0 |  |
|  | Anoxic (7) | 0.98 | 0 | 0.01 | 0.01 | 7963 ± 923 |
| Melb. Beach (surface) | Oxic (0) | 0.49 | 0.30 | 0.20 | 0.01 |  |
|  | Anoxic (0) | 0.23 | 0.05 | 0.66 | 0.07 |  |
|  | Anoxic (1) | 0.25 | 0 | 0.74 | 0.01 |  |
|  | Anoxic (7) | 0.84 | 0 | 0.16 | 0 | 838 ± 228 |
| Melb. Harbour (surface) | Oxic (0) | 0.57 | 0 | 0.24 | 0.19 |  |
|  | Anoxic (0) | 0.35 | 0.04 | 0.60 | 0 |  |
|  | Anoxic (1) | 0.42 | 0 | 0.57 | 0 |  |
|  | Anoxic (7) | 0.60 | 0 | 0.38 | 0.02 | 1937 ± 1061 |
| Fӕllesstrand (surface) | Oxic (0) | 0.74 | 0.06 | 0.20 | 0 |  |
|  | Anoxic (0) | 0 | 0 | 0.93 | 0.07 |  |
|  | Anoxic (1) | 0.86 | 0 | 0.10 | 0.04 |  |
|  | Anoxic (7) | 0.90 | 0 | 0.10 | 0 | 3173 ± 613 |
| Hjerting Beach (surface) | Oxic (0) | 0.60 | 0.07 | 0.32 | 0 |  |
|  | Anoxic (0) | 0.38 | 0.05 | 0.55 | 0.02 |  |
|  | Anoxic (1) | 0.64 | 0.01 | 0.35 | 0 |  |
|  | Anoxic (7) | 0.96 | 0 | 0.02 | 0.02 | 3638 ± 999 |
| Melb. Beach (deep) | Oxic (0) | 0.56 | 0.12 | 0.18 | 0.14 |  |
|  | Anoxic (0) | 0.41 | 0.01 | 0.58 | 0 |  |
|  | Anoxic (1) | 0.46 | 0.04 | 0.50 | 0 |  |
|  | Anoxic (7) | 0.83 | 0 | 0.17 | 0 | 150 ± 85 |
| Melb. Harbour (deep) | Oxic (0) | 0.64 | 0 | 0.28 | 0.07 |  |
|  | Anoxic (0) | 0.60 | 0 | 0.40 | 0 |  |
|  | Anoxic (1) | 0.88 | 0 | 0.12 | 0 |  |
|  | Anoxic (7) | 0.01 | 0 | 0.93 | 0.06 | 1360 ± 801 |
| Fӕllesstrand (deep) | Oxic (0) | 0.72 | 0.02 | 0.26 | 0 |  |
|  | Anoxic (0) | 0.30 | 0.01 | 0.67 | 0.02 |  |
|  | Anoxic (1) | 0.85 | 0 | 0.14 | 0 |  |
|  | Anoxic (7) | 0.96 | 0.01 | 0.02 | 0 | 1206 ± 341 |
| Yarra | Oxic (0) | 0.81 | 0 | 0.18 | 0.01 |  |
|  | Anoxic (0) | 0.88 | 0 | 0.12 | 0 |  |
|  | Anoxic (1) | 0.84 | 0 | 0.16 | 0 |  |
|  | Anoxic (7) | 0.77 | 0 | 0.23 | 0 | 2752 ± 573 |
| Gippsland | Oxic (0) | 0.51 | 0.18 | 0.31 | 0 |  |
|  | Anoxic (0) | 0.71 | 0.02 | 0.27 | 0 |  |
|  | Anoxic (1) | 0.91 | 0 | 0.09 | 0 |  |
|  | Anoxic (7) | 0.79 | 0 | 0.21 | 0 | 535 ± 478 |
| Patterson | Oxic (0) | 0.66 | 0 | 0.33 | 0.01 |  |
|  | Anoxic (0) | 0.36 | 0 | 0.63 | 0.01 |  |
|  | Anoxic (1) | 0.43 | 0.04 | 0.53 | 0 |  |
|  | Anoxic (7) | 0.88 | 0 | 0.12 | 0 | 3846 ± 737 |

**Supplementary Table S4.** Oxygen concentrations at the FTR outlet at each timepoint.

| Time (hours) | Oxygen concentration (µmol L^-1^) |
| --- | --- |
| 0 | 60 |
| 2 | 35 |
| 8 | 22 |
| 11 | 15 |
| 18 | 0 |
| 28 | 0 |
| 46 | 0 |
| 98 | 0 |
| 148 | 0 |

**Supplementary Table S5.** *R_1_* values and fraction of fermentation ${(f}_{EMP ferm}$) at each timepoint during FTR experiment.

| Time (hours) | R_1_ values | $f_{EMP ferm}$ values |
| --- | --- | --- |
| 0 | 0.22 | 0 |
| 4 | 0.14 | 0.18 |
| 10 | 0.14 | 0.16 |
| 19 | 0.24 | 0 |
| 28 | 0.08 | 0.53 |
| 48 | 0.05 | 0.71 |
| 66 | 0.05 | 0.69 |
| 98 | 0.11 | 0.36 |
| 122 | 0.12 | 0.26 |
| 147 | 0.12 | 0.31 |
| 166 | 0.13 | 0.21 |

**Supplementary Table S6.** The relative contribution of respiration, ED fermentation, EMP fermentation and PP fermentation to each pure culture incubation as determined using the Rsolnp R package (see **Calculations**).

| **Bacteria** | **Incubation** | **Resp.** | **PP ferm.** | **ED ferm.** | **EMP ferm.** |
| --- | --- | --- | --- | --- | --- |
| *Lutibacter* | Oxic | 0.08 | 0 | 0.02 | 0.9 |
|  | Anoxic | 0.01 | 0.06 | 0 | 0.93 |
| *Vibrio* | Oxic | 0.27 | 0 | 0.15 | 0.58 |
|  | Anoxic | 0 | 0.39 | 0 | 0.61 |
| *Tropicimonas* | Oxic | 0.5 | 0 | 0.31 | 0.19 |
|  | Anoxic | 0.27 | 0 | 0.03 | 0.7 |
| *Maribacter* | Oxic | 0.08 | 0 | 0.02 | 0.9 |
|  | Anoxic | 0.05 | 0 | 0.05 | 0.9 |

**Supplementary Table S7.** Concentrations of volatile fatty acids (VFA) (µM) including lactate, acetate, formate, propionate, iso-butyrate, butyrate, succinate, iso-valerate and valerate under oxic and anoxic conditions for *Lutibacter* and *Maribacter*.

|  | **Concentrations (µM)** | | | | | | | | | |
| --- | --- | --- | --- | --- | --- | --- | --- | --- | --- | --- |
|  | Lactate | Acetate | Formate | Propionate | Iso-butyrate | Butyrate | Succinate | Iso-valerate | Valerate | **Total** |
| ***Lutibacter*** | | | | | | | | | | |
| Oxic 2 days | 0 | 257 ± 60 | 103 ± 30 | 562 ± 200 | 0 | 429 ± 100 | 98 ± 20 | 543 ± 80 | 0 | 1992 ± 200 |
| Oxic 4 days | 0 | 424 ± 80 | 1213 ± 700 | 367 ± 200 | 3847 ± 400 | 456 ± 300 | 41 ± 30 | 538 ± 400 | 26 ± 30 | 6911 ± 1000 |
| Anoxic 2 days | 0 | 115 ± 40 | 0 | 0 | 0 | 309 ± 100 | 91 ± 50 | 42 ± 50 | 0 | 557 ± 100 |
| Anoxic 4 days | 106 ± 90 | 461 ± 200 | 93 ± 40 | 690 ± 300 | 0 | 382 ± 100 | 342 ± 80 | 41 ± 70 | 0 | 2115 ± 400 |
| ***Maribacter*** | | | | | | | | | | |
| Oxic 2 days | 0 | 32 ± 30 | 194 ± 30 | 940 ± 200 | 0 | 382 ± 200 | 0 | 390 ± 200 | 64 ± 20 | 2002 ± 400 |
| Oxic 4 days | 0 | 4421 ± 4000 | 748 ± 200 | 1168 ± 300 | 5536 ± 1678 | 719 ± 300 | 0 | 2375 ± 300 | 166 ± 100 | 15132 ± 4000 |
| Anoxic 2 days | 84 ± 60 | 359 ± 100 | 85 ± 30 | 195 ± 100 | 0 | 163 ± 100 | 385 ± 200 | 0 | 0 | 1270 ± 200 |
| Anoxic 4 days | 121 ± 200 | 996 ± 500 | 177 ± 80 | 1241 ± 500 | 0 | 131 ± 200 | 630 ± 100 | 0 | 0 | 3296 ± 700 |
