# Supplementary figures and images for: Microorganisms oxidize glucose through distinct pathways in permeable and cohesive sediments
Source: ISME J. 2024 Jan 30;18(1):wrae001. doi: 10.1093/ismejo/wrae001 (PMC10939381; doi:10.1093/ismejo/wrae001)

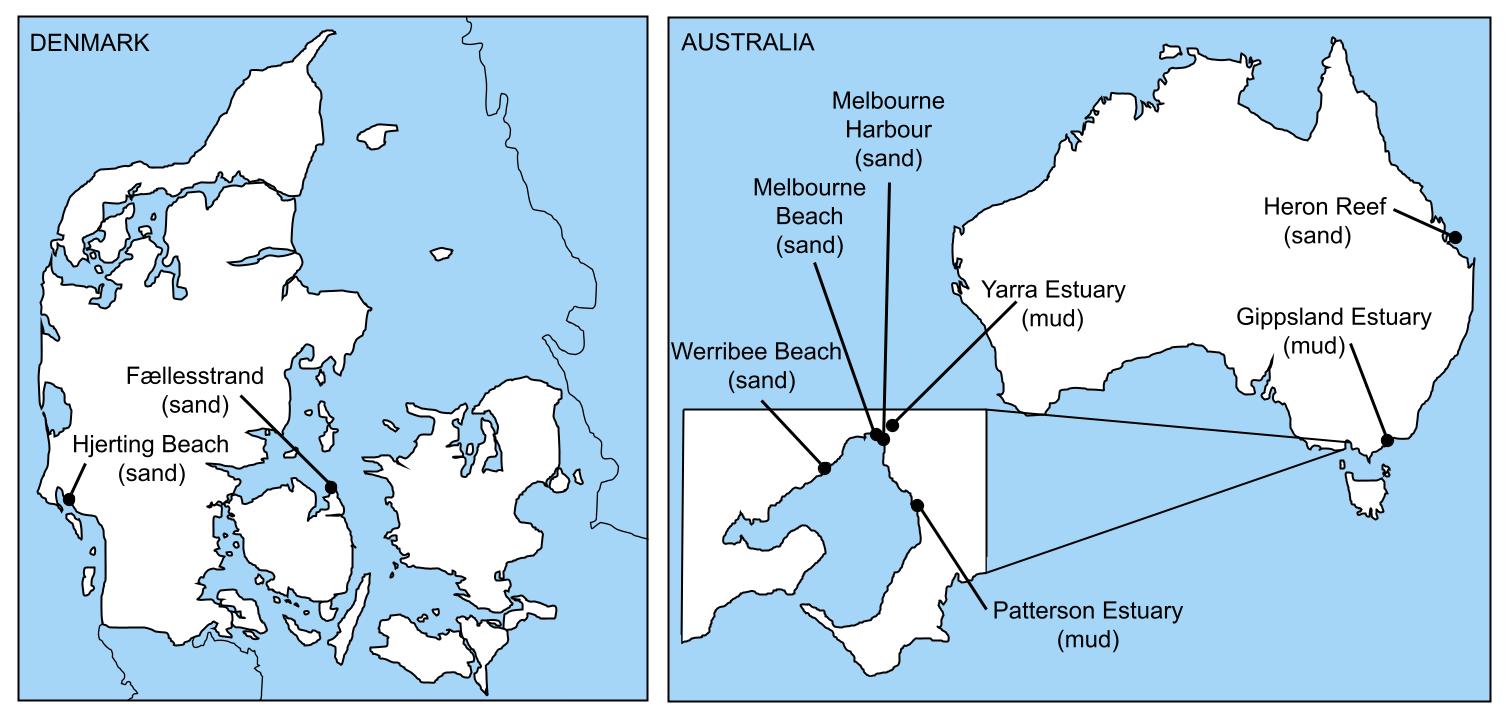

Supplement: supplementary_material_wrae001 [file supplementary_material_wrae001.zip › Fig S1.jpg]

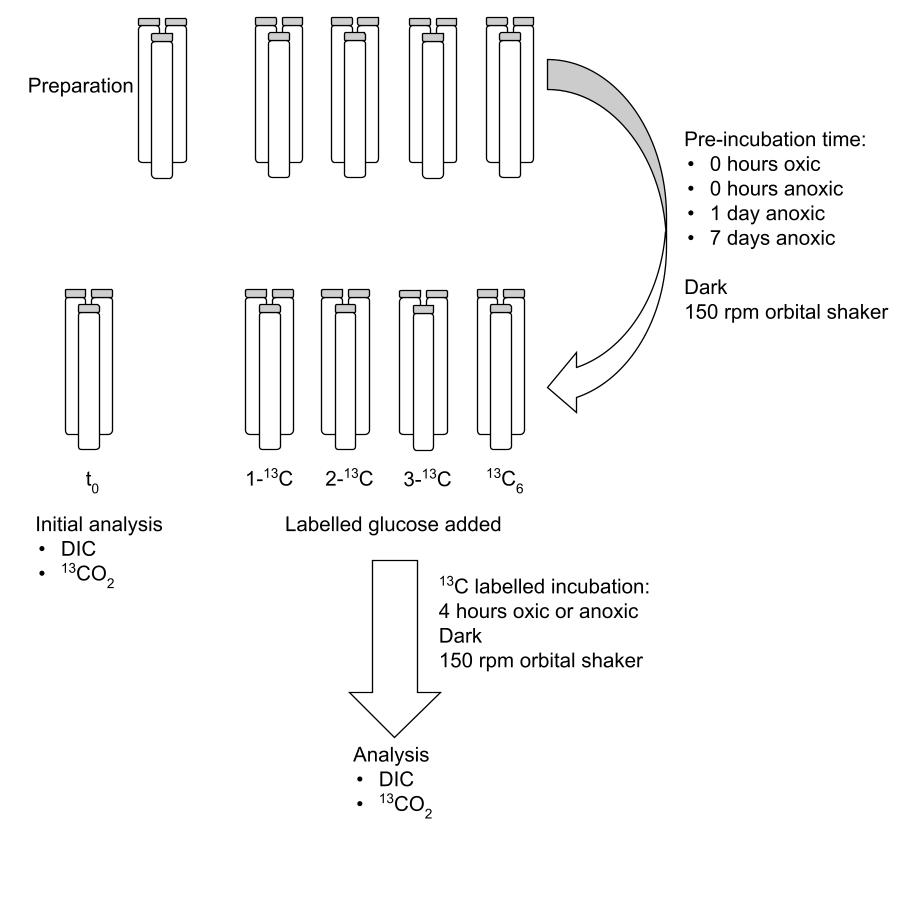

Supplement: supplementary_material_wrae001 [file supplementary_material_wrae001.zip › Fig S2.jpg]

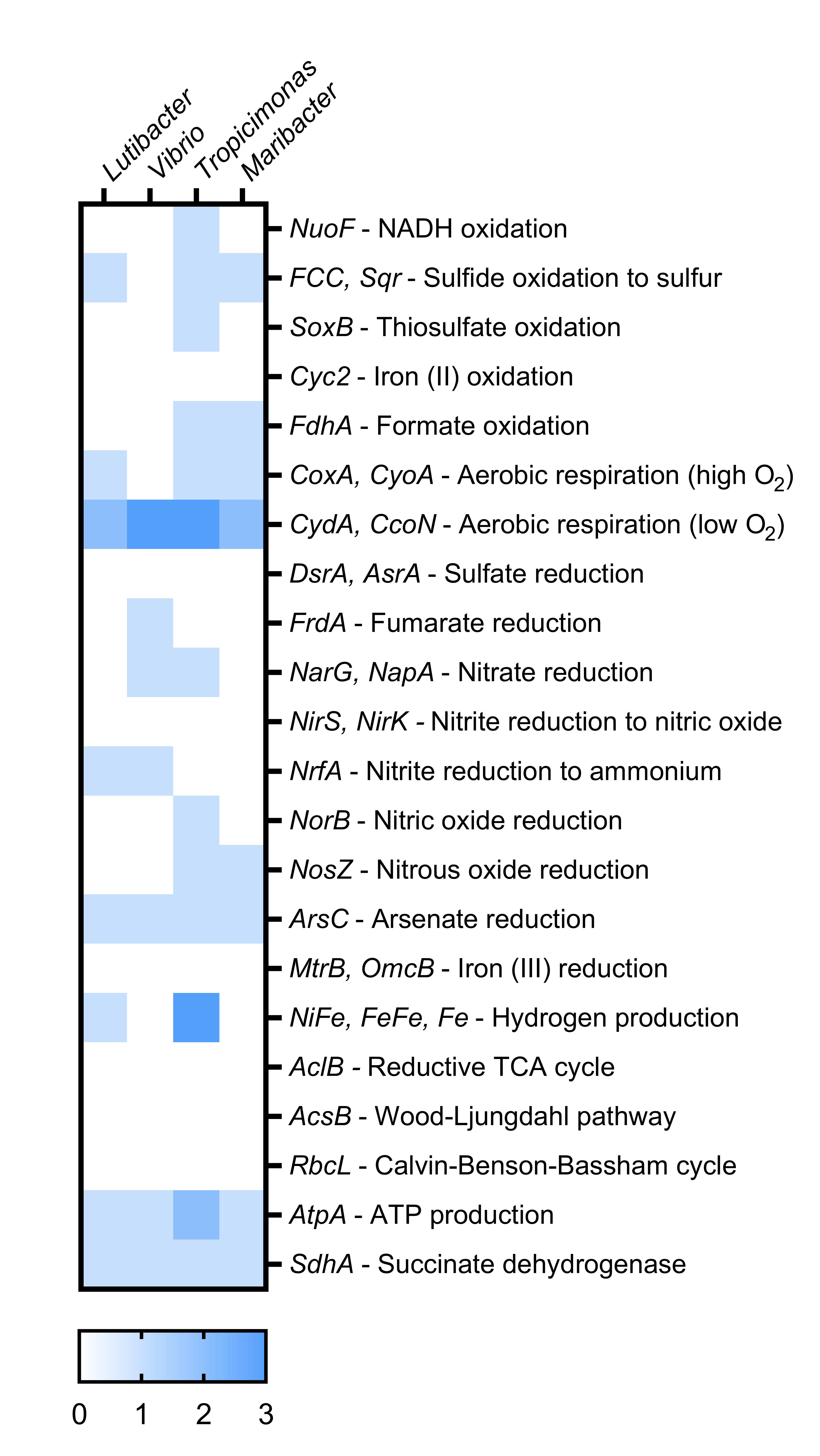

Supplement: supplementary_material_wrae001 [file supplementary_material_wrae001.zip › Fig S5.jpg]
